# Spatiotemporal dynamics of a glioma immune interaction model

**DOI:** 10.1038/s41598-021-00985-1

**Published:** 2021-11-17

**Authors:** Subhas Khajanchi, Juan J. Nieto

**Affiliations:** 1 Department of Mathematics, Presidency University, 86/1 College Street, Kolkata, 700073 India; 2 Instituto de Matem\acute{a}ticas, Universidade de Santiago de Compostela, 15782 Santiago de Compostela, Spain

**Keywords:** Cancer, Medical research, Oncology, Mathematics and computing

## Abstract

We report a mathematical model which depicts the spatiotemporal dynamics of glioma cells, macrophages, cytotoxic-T-lymphocytes, immuno-suppressive cytokine TGF-β and immuno-stimulatory cytokine IFN-γ through a system of five coupled reaction-diffusion equations. We performed local stability analysis of the biologically based mathematical model for the growth of glioma cell population and their environment. The presented stability analysis of the model system demonstrates that the temporally stable positive interior steady state remains stable under the small inhomogeneous spatiotemporal perturbations. The irregular spatiotemporal dynamics of gliomas, macrophages and cytotoxic T-lymphocytes are discussed extensively and some numerical simulations are presented. Performed some numerical simulations in both one and two dimensional spaces. The occurrence of heterogeneous pattern formation of the system has both biological and mathematical implications and the concepts of glioma cell progression and invasion are considered. Simulation of the model shows that by increasing the value of time, the glioma cell population, macrophages and cytotoxic-T-lymphocytes spread throughout the domain.

## Introduction

According to the report from National Brain Tumor Society^1^, it was estimated about 700,000 people in the USA are living with primary brain tumor, and 84,170 people will be diagnosed with primary brain tumor among which 59,040 people are non-malignant (benign) and 25,130 are malignant brain tumor and 18,600 deaths are projected to occur in the year 2021. Brain tumor is a world-wide problem, which can be regarded as one of the major causes of death but still a mystery about its mechanism of growth, prevention and cure. Brain tumor comprises a heterogeneous group of intracranial neoplasms and it is difficult to treat due to its sequestered location beyond the blood-brain-barrier and their infiltrative behavior^2^. More than 120 different kinds of tumor can be found in the human brain; among them glioblastoma is the most common type of primary brain tumor. It has median survival rate ranging from 12 to 14 months from the time of diagnosis^3^. The most aggressive or grade IV gliomas are known as glioblastoma multiforme (GBM), which is the main goal of our study. According to the 2016 World Health Organization (WHO) grading scheme for GBM of the Central Nervous System, the GBM is mainly classified into (i) isocitrate dehydrogenase (IDH)-wildtype glioblastoma (nearly 90% of cases) that predominates in the patients over 55 years of age; (ii) IDH-mutant glioblastoma (nearly 10% of cases) that preferentially arises in younger patients^4^. Most of the treatments like standard chemotherapy, surgery, radiation therapy have limited success due to the heterogeneity of gliomas, infiltrative behavior, genomic instability including the presence of blood-brain-barrier. Brain neoplasms may be explained as an irregular growth of normal tissues than its surrounding normal brain tissues. The interaction between malignant gliomas and immune system is a nonlinear and highly complex phenomena. This nonlinearity has attracted the attention of a significant number of scientists and researchers/oncologists in investigating the dynamics of glioma-immune system interactions throughout the world.

To better understand such complex phenomenon, researchers have used mathematical models of glioma-immune system response, over the last few decades. The mathematical modeling is one of the important tools which provide realistic, quantitative and qualitative representations of important biological phenomena. The biological interpretation obtained from mathematical models give the realistic predictions of the state of brain tumors under different situations^5^. A variety of mathematical models has been proposed by numerous authors (see the review article^6^) to understand the complex biological process and to design better treatment strategies or to improve the patients’ quality of life. Immunotherapy has become a quickly developing process in glioma treatment, based on the assumption that the immune system can be strengthened to combat against gliomas. Various immunotherapeutic treatments have been developed in aiding the immune system in efficient identification and eradication of glioma cells^7^.

There are some interesting literatures, which described the temporal dynamics of tumor growth in presence of immune system interaction through a system of ordinary differential equations^8–13^. Spatiotemporal pattern formation in reaction-diffusion equations has been studied continuously and become an important issue to the researchers due to the pioneering work by Turing 1952^14^. After that a significant number of mathematical models for tumor growth have been developed and the application of these models have been increased recently by using a system of reaction-diffusion equations^6,15^. Different kinds of brain tumor models have already been developed by the researchers, and each one contributes in its own way to a better understanding of brain tumor and the complicated dynamics that delineate the patient’s outcome^16–20^. Numerous articles have been focused on the mathematical modeling of gliomas environment in various aspects through a system of differential equations to put forward the theoretical models for glioma immunology and immunotherapy or chemotherapy^21–32^. A series of work has been done on the basis of pioneering mathematical model for brain tumor developed by Swanson et al.^5,20,33,34^ and Murray^35^. Swanson et al.^5,20,25,33,34,36^ investigated the mathematical models that have been developed by Murray^35^ and herself. Their models quantify the spatiotemporal proliferation and invasion of malignant gliomas in the virtual human brain.

Celiku et al.^26^ investigated a computational model by introducing new concepts of phenotype to delineate probable spatial-phenotypic trajectories on the basis of patient data. Engwer and Wenske^27^ studied a advection-diffusion-reaction equations to understand the complicated dynamics of glioblastoma invasion at the time of a given MRI/DTI scan by utilizing the stationalized approach. Corbin et al.^28^ studied a mathematical model for glioma invasion by considering the dynamics of brain tissues being actively degraded by glioma cells through excessive production of acidity. Their method admits switching phenomena between slow and faster moving regimes based on the local tissue anisotropy. Perrillat-Mercerot et al.^29^ studied a mathematical model for the growth of glioma cells that describes the modeling history, attentiveness and the limitations of the study of glioblastomas. In^30^, the authors developed a mathematical model for glioblastomas by assuming that the immune cells migration to the glioma site along with chemotactic gradient field. The authors performed numerical simulations based on the experimental data. In^31^, the authors studied a mathematical model as an alternative therapeutics for glioma cell population by combining the chemotherapy and oncolytic virotherapy. Conte et al.^32^ proposed a multiscale mathematical model for the migration of glioma cells and proliferation by considering the role of therapeutics. Swanson et al.^33^ studied a mathematical model for drug delivery of chemotherapeutic agents to treat malignant gliomas. In the review article^5^, the authors investigated a mathematical model for glioma proliferation and aggressiveness with and without treatment strategy through a system of reaction-diffusion equations. Kirkby et al.^37^ developed a very generic experimentally validated phenomenological mathematical model for the proliferation and invasion of aggressive brain tumors. They used Monte Carlo simulation to fit the model with clinical investigations.

Owen and Sherratt^15^ proposed a mathematical model through a system of reaction-diffusion equations by considering the spatial interplays for macrophages, mutant cells and the normal cells, indicating the capability of macrophages to destroy the tumor cells. Habib et al.^24^ studied a theoretical and numerical framework of a continuum model for brain tumor consists of six reaction-diffusion system to describe chemotactic and haptotactic cell dynamics. Tanaka et al.^38^ constructed a hybrid compartment-continuum-discrete model with a goal to investigate the progression and infiltration of malignant gliomas. Kim et al.^22^ developed a mathematical model to describes the dynamics of glioma cells in presence of immune responses using a reaction-diffusion system by introducing the effect of adhesion and gives an elucidation for the different patterns of cell migration. Later on, Kim et al.^23^ constructed another mathematical model of high-grade glioblastoma evolution that concentrated on the relative balance of the migration and proliferation of glioma cells. Their model gives an interpretation for the growth-invasion cycling patterns of glioma cell population in response to high/low glucose uptake in microenvironment. Toma et al.^39^ investigated a mathematical model that determine the proliferation and infiltration of malignant gliomas at a cellular level by introducing the role of microglia/macrophages cells with the help of reaction-diffusion equations.

In the present paper we address a mathematical model that describes the interplay between malignant gliomas, macrophages, cytotoxic T-lymphocytes, immuno-stimulatory cytokine IFN-γ and the immuno-suppressive cytokine TGF-β, through a coupled system of reaction-diffusion equations. The main aim of this article is to gain an insight into mechanisms of spatiotemporal pattern formation in a glioma-immune interaction model. We investigate the heterogeneous nature of high-grade gliomas, macrophages, cytotoxic T-lymphocytes in both one and two dimensional spaces. The outcomes of our computational studies highlight a wide range of spatiotemporal dynamics, most important of which are patterns of spatiotemporal heterogeneity. Interestingly, the investigated spatiotemporal heterogeneity is mainly caused by the interplay of the cell populations in space and time which indicates the phenomena of long-term dynamics of the glioma-immune interaction.

The organization of this article is as follows. After the introduction, we described the glioma-immune interaction model and their microenvironment through a reaction-diffusion system. In the “Qualitative study the model”, we analyze the mathematical model and state the conditions for local asymptotic stability without diffusion, then investigate the model system with diffusion coefficient. In the same section, we observed that the temporally stable interior equilibrium point $E^{*}$ remains stable under the small inhomogeneous spatiotemporal perturbations. In the “Sensitivity analysis”, sensitivity analysis is studied using Partial Rank Correlation Coefficient (PRCC) to identify which system parameters are most effective with respect to the model output. In the “Spatiotemporal dynamics”, we presented some numerical simulations in one and two-dimensions. Finally we discuss the main outcomes of the present analysis and propose future directions of our research.

## The spatiotemporal model

Our aim in the creation of a model system is to allow sufficient complexity so that the model will qualitatively generate clinically observed in vivo malignant gliomas growth patterns, while it simultaneously maintains enough simplicity to admit model analysis. Our previous mathematical model that we modified here^16^ depicts the proliferation of glioma cell population and immune system interaction through a system of coupled nonlinear ordinary differential equations by considering the effect of immunotherapeutic drug T11 target structure. However, the work was significant but simple in the sense that the authors did not take into account the movement of malignant gliomas and immune system responses. The glioma cells are highly diffusive and invasive in nature and its survival rate varies from 1 year to 3 years in the category of Grade IV and Grade III, respectively^3,36^. Also, the glioma cells are the heterogeneous group intracranial neoplasms, undifferentiated and genomically unstable^5^.

Different factors influence glioma cell migration in the brain. Migration can be stimulated by extracellular matrix (ECM) in a process known as haptotactic migration^22^. Glioma cells motility is also stimulated by different molecules which they secrete, leading to chemotactic migration^23^. Despite progresses in understanding the rudimentary biology of glioblastoma, studies on glioma cell migration are hindered due to the lack of efficient in vitro or in vivo migration models. Migration of human glioma stem cells occurs in the brain parenchyma through continuous interplay with axon fibers, glial cells, microglia, and endothelial cells that likely affect their migratory efficiency^40,41^. Glioma cells have been expressed to respond to a variety of migratory cues, incorporating chemotactic, galvanotactic as well as mechanical cues^42,43^. These migratory cues are being actively investigated to promote the understanding of glioblastoma migratory behavior and to identify possible methods of intervention.

In patients, glioma cells usually follow preferred dispersion routes such as along white matter tracts and the basal lamina of brain blood vessels. This indicates that glioma cell migration may be dependent on specific substrates and structures in the brain. There are some existing literatures which focused on the spatiotemporal dynamics of glioma-immune interactions to better understand the invasion of malignant gliomas and immune system interplays in the brain of patients^5,20–25,27–30,32–34,36,38,44–47^. Altogether it is clear that the overall extent and pattern of invasion is due to multiple factors including the inherent properties of the glioma cells themselves and their microenvironment.

**Figure 1:**
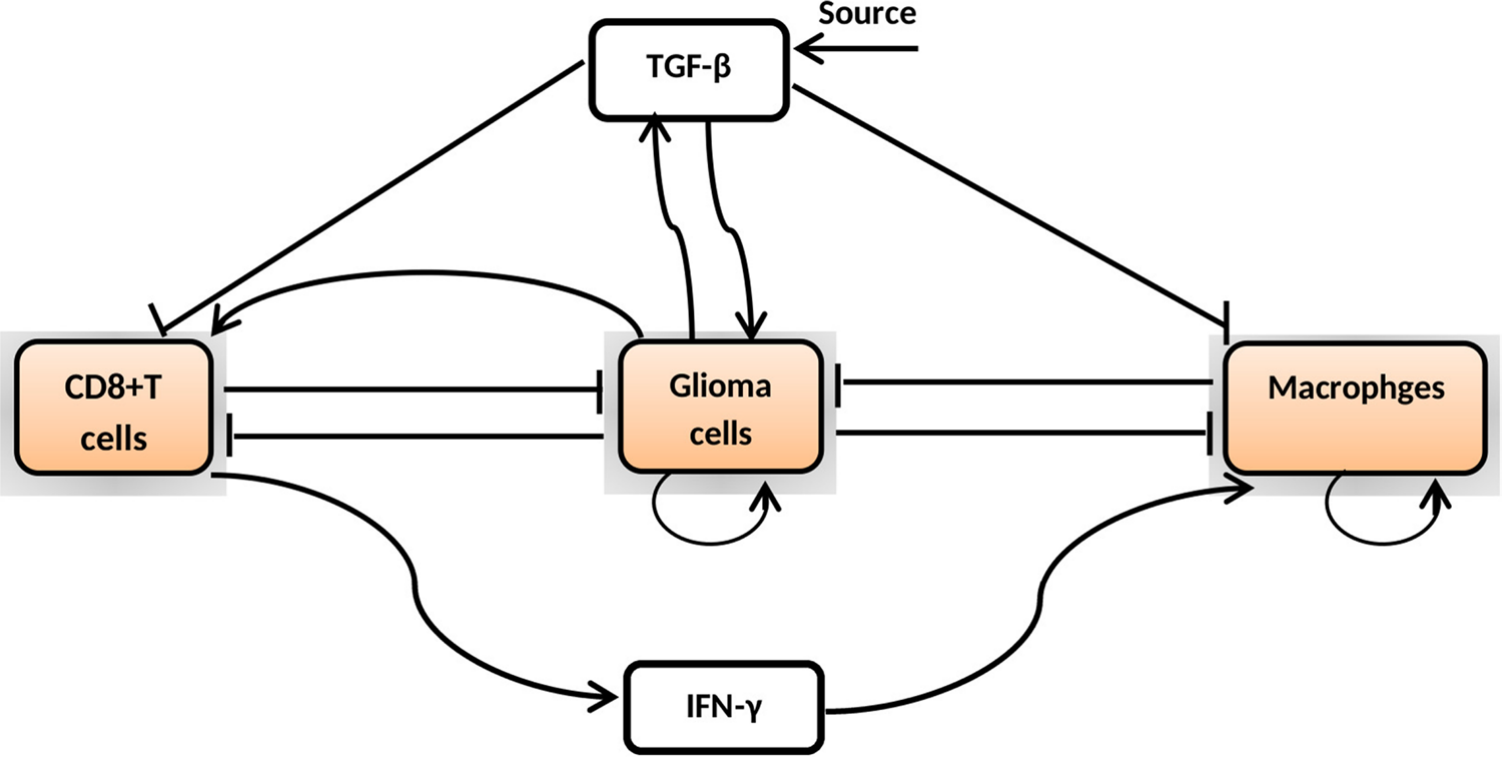
A schematic diagram represents the interaction of cells and cytokines that are used in the model. The model represents cells (colored rectangles) and cytokines (clear rectangles). Sharp arrows indicate proliferation/activation, blocked arrows indicate killing/blocking/inhibition and round arrows indicate self-proliferation of cells.

We present a schematic diagram for the glioma-immune interaction model in Fig. 1 that describes the proliferation and inhibition of cell populations as well as the interactions between all model species. The glioma-immune interaction model itself is presented in Eqs. (1)–(5), which we have interpreted to give a full description of each of the terms in the model.

The right hand side of Eq. (1) describes the growth of malignant gliomas without any immune responses, using logistic growth function where *G* represents glioma cell numbers at any moment. The term $r_{1}$ represents the proliferation rate and $G_{\max }$ stands for the carrying capacity or maximum glioma cell burden. The second term of (1) designates the eradication of glioma cells by the immune components macrophages (*M*) and cytotoxic T-lymphocytes $(C_{T})$ at the rates $\alpha _{1}$ and $\alpha _{2}$, respectively. The immuno-suppressive factor TGF-$\beta$ secreted by malignant gliomas^48^ has tried to down-regulate the activity of both the immuno-stimulatory components macrophages and activated cytotoxic T-lymphocytes^49^. The final term describes the random motility of malignant glioma cells where $D_G$ represents the diffusion coefficient. Combining these assumption gives the following reaction-diffusion equation for malignant gliomas:

1 $$\begin{aligned} \frac{\partial G}{\partial t}= \, & {} \overbrace{r_{1}G\left( 1-\frac{G}{G_{\max }}\right) }^{logistic~growth}-\overbrace{\frac{(\alpha _{1}M + \alpha _{2} C_{T})G}{(e_{1}+T_{\beta })(G+k_{1})}}^{degradation ~of~gliomas} + \overbrace{D_{G} \nabla ^{2} G}^{random~motility}. \end{aligned}$$

Macrophages are one of the crucial innate immune cells associated in the regulation of anti-cancer immunotherapies, having either immuno-stimulatory or immunosuppressive effects^50^. The heterogeneity of macrophages, with phenotypes varying from the anti-tumor classically-activated M1 (pro-inflammatory) macrophages to the pro-tumor alternatively-activated M2 (anti-inflammatory) macrophages, makes it hard to understand and progression and control of tumor cell population^51^. M1 macrophages are activated by IFN-γ, and secrete high levels of IL-12 and low levels of IL-10 whereas M2 macrophages produce high levels of IL-10, TGF-β and low levels of IL-12. Tumor-associated macrophages are mainly M2 phenotype, and actively promote growth of tumor cells^50,51^. In our study, we have considered M1 phenotype that is mainly activated by IFN-γ and suppresses the activity of glioma cell population^52^.

The macrophage populations are highly heterogeneous in nature, in terms of its activation/proliferation and chemotaxis^53^, and so one can predict a more extensive set of variables that would include numerous subpopulations of macrophages. The macrophages population are able to move through pseudopodia; so our assumption in the previous paper^16^ was unrealistic in the sense that the activation of macrophages are pointwise in space. In order to overcome this restriction, we introduce the movement of macrophages through a reaction-diffusion system.

We assume that macrophages can proliferate logistically with intrinsic growth rate $r_{2}$ and maximum carrying capacity $M_{\max }$ due to crowding effect of all cell types. The macrophages cell populations are activated by the immuno-stimulatory cytokine IFN-γ at the rate $a_{1}$, and at the same time it is down-regulated by immuno-suppressive cytokine TGF-β. We assume that the cell count of macrophages decrease due to the interaction with glioma cell population at the rate $\alpha _{3}$ and their interaction follow the Michaelis–Menten saturation dynamics with $k_{2}$ being a half saturation constant. The Michaelis–Menten kinetics is described by a linear denominator with the constant $k_{2}$ representing the accessibility of the glioma cells to macrophages. So that the conversation equation for macrophages population is given by

2 $$\begin{aligned} \frac{\partial M}{\partial t}= \, & {} \overbrace{r_{2}M\left( 1-\frac{M}{M_{\max }}\right) }^{logistic~growth}+ \overbrace{a_{1}\left( \frac{I_{\gamma }}{k_{4}+I_{\gamma }}\right) }^{proliferation} \underbrace{\left( \frac{1}{T_{\beta }+e_{2}}\right) }_{down-regulation} -\overbrace{\alpha _{3}\frac{G M}{k_{2}+ G}}^{inactivation~term}+\overbrace{D_{M} \nabla ^{2} M}^{random~motility}. \end{aligned}$$

Now, we study the kinetics of activated cytotoxic T-lymphocytes and other cell population as well as the mechanisms of migration and the diffusion of cytotoxic T-lymphocytes^54^. In our study, we considered activated cytotoxic T-lymphocytes as the activated cytotoxic T-lymphocytes can cross the blood-brain-barrier and gain entry into the brain^18^. It is important to take into account that there is no ‘nonlinear’ movement of cytotoxic T-lymphocytes and no ‘nonlinear’ diffusion of chemokine, that is, all the random motility and diffusion terms are supposed to be constant^55^.

We consider that the proliferation of cytotoxic T-lymphocytes occur due to direct presence of malignant glioma cell population, where the parameter $a_{2}$ models the rate of antigenicity of malignant gliomas. Antigenicity can be considered as a measure of how different the gliomas are from ‘self’. The recruitment of cytotoxic T-lymphocytes is inhibited by immunosuppressive cytokine TGF-β where $k_{5}$ is termed as an inhibitory parameter. Cytotoxic T-lymphocytes have a lifespan on an average $1/\mu _{1}$ days. The clearance of cytotoxic T-lymphocytes by malignant gliomas occur through the Michaelis-Menten saturation dynamics at the rate $\alpha _{4}$ and the half saturation constant $k_{3}$. Michaelis-Menten form represents the saturated effects of the immune response due glioma cell population. Therefore, the reaction-diffusion equation governing the evolution of cytotoxic T-lymphocytes density is

3 $$\begin{aligned} \frac{\partial C_{T}}{\partial t}= \, & {} \overbrace{a_{2}G}^{recruitment}. \underbrace{\frac{1}{k_{5}+T_{\beta }}}_{inhibition} \overbrace{- \mu _{1} C_{T}}^{natural~decay} - \overbrace{\alpha _{4}\frac{G}{k_{3}+ G}C_{T} }^{inactivation~term} + \overbrace{D_{C} \nabla ^{2} C_{T}}^{diffusion}. \end{aligned}$$

Transforming growth factor β (TGF-β) signaling is related to the regulation of proliferation, differentiation and survival (or apoptosis), among several type of cells including glioma cells^7^. The glioma induced cytokines TGF-β, prostaglandin $E_{2}$ and IL-10 suppress the activity of immune system and stimulate the production of malignant gliomas. When the gliomas are sufficiently small it secrete small amount of TGF-β to obtain ample nutrients from the neighboring tissue. However, as the glioma cell population grows sufficiently large, glioma cells suffer from the lack of oxygen, nutrients and space, it begins to produce TGF-β to stimulate angiogenesis and to invade the immune response once glioma growth resumes^48,56^.

TGF-β has a constant source rate $s_{1}$ in central nervous system (CNS). Production of TGF-β is proportional to the size of glioma cell population, at the rate $b_{1}$. TGF-β is also supposed to decay linearly at the rate $\mu _{2}$. We assume that TGF-β diffuse throughout the tissue with a slower rate than glioma cell population. Therefore, the reaction-diffusion equation for the density of TGF-β is given by

4 $$\begin{aligned} \frac{\partial T_{\beta }}{\partial t}= \, & {} \overbrace{s_{1} + b_{1}G}^{growth~kinetics} - \overbrace{ \mu _{2} T_{\beta }}^{decay} + \overbrace{ D_{T} \nabla ^{2} T_{\beta }}^{diffusion}. \end{aligned}$$

Interferon-γ (IFN-γ) is a cytokine acting on cell-surface receptors and activated transcription of genes. IFN-γ is produced by cytotoxic T-lymphocytes in the glioma micro-environment^57^. The dynamics of IFN-γ is depicted in Eq. (5). First term on the right side of (5) represents the linear production of IFN-γ and $b_{2}$ represents the release rate of a single cytotoxic T-lymphocytes. We consider that the source of IFN-γ is cytotoxic T-lymphocytes^18^. The density of IFN-γ is also assumed to decay linearly at the rate $\mu _{3}$. We assume that the immuno-stimulatory cytokine IFN-γ diffuse throughout the tissue within the brain. Hence, the reaction-diffusion equation governing the evolution of the IFN-γ is

5 $$\begin{aligned} \frac{\partial I_{\gamma }}{\partial t}= \, & {} \overbrace{b_{2}C_{T}}^{production} - \overbrace{\mu _{3}I_{\gamma }}^{decay} + \overbrace{D_{I} \nabla ^{2} I_{\gamma }}^{diffusion}, \end{aligned}$$

where $\nabla ^{2} \left( \equiv \frac{\partial ^{2}}{\partial x^{2}} + \frac{\partial ^{2}}{\partial y^{2}} \right)$ represents the two-dimensional Laplacian operator, subject to the zero-flux boundary conditions with the given nonnegative initial distribution of glioma cell population (*G*), macrophages (*M*), activated cytotoxic T-lymphocytes $(C_{T})$, cytokine TGF-β $(T_{\beta })$ and the cytokine IFN-γ $(I_{\gamma })$ are given by

6 $$\begin{aligned} \frac{\partial G}{\partial n}= \, & {} \frac{\partial M}{\partial n} = \frac{\partial C_{T}}{\partial n} = \frac{\partial T_{\beta }}{\partial n} = \frac{\partial I_{\gamma }}{\partial n} = 0~~~\text {on}~~\partial \Omega ,~~~t~>~0, \end{aligned}$$

7 $$\begin{aligned} G(x,y,0)= \, & {} G_{0}(x,y)> 0,~ M(x,y,0) = M_{0}(x,y)> 0, ~ C_{T}(x,y,0) = C_{T0}(x,y)> 0, \nonumber \\ T_{\beta }(x,y,0)= \, & {} T_{\beta 0}(x,y)> 0, ~I_{\gamma }(x,y,0) = I_{\gamma 0}(x,y) > 0,~~~\text {for}~~~(x, y) \in \Omega . \end{aligned}$$

Here $\Omega$ represents the bounded square domain (simply connected) with boundary $\partial \Omega$, $\partial /\partial n$ indicates outward drawn normal derivative on $\partial \Omega$ with $D_{G}$, $D_{M},$ $D_{C}$, $D_{T}$ and $D_{I}$ are the diffusion coefficients for glioma cell population (*G*), macrophages (*M*), CD8+T cells $(C_{T})$, TGF-$\beta$ $(T_{\beta })$ and IFN-γ $(I_{\gamma })$, respectively. In the model of Eqs. (1)–(5), *G*(*x*, *y*, *t*), *M*(*x*, *y*, *t*), $C_{T}(x, y, t)$, $T_{\beta }(x, y, t)$ and $I_{\gamma }(x, y, t)$ represents the densities glioma cells, macrophages, CD8+ T cells, TGF-β and IFN-γ respectively, at time *t* with $(x, y) \in \Omega$ is the position in space.

## Qualitative study the model

In this section, we shall investigate the spatially homogeneous steady states of the glioma-immune interaction system (1)–(5) and their local stability analysis. In order to do that, we consider $D_{G} ~= D_{M} ~= D_{C} ~= D_{T} ~= D_{I} ~= 0$ and the reaction-diffusion model system (1)–(5) reduces to the temporal system as follows

8 $$\begin{aligned} \frac{d G}{d t}= \, & {} r_{1}G\left( 1-\frac{G}{G_{\max }}\right) -\frac{(\alpha _{1}M + \alpha _{2} C_{T})G}{(e_{1}+T_{\beta })(G+k_{1})} ~\equiv f_{1}(G, M, C_{T}, T_{\beta }, I_{\gamma }), \nonumber \\ \frac{d M}{d t}= \, & {} r_{2}M\left( 1-\frac{M}{M_{\max }}\right) +a_{1}\left( \frac{I_{\gamma }}{k_{4}+I_{\gamma }}\right) \left( \frac{1}{T_{\beta }+e_{2}}\right) -\alpha _{3}\frac{G M}{k_{2}+ G} ~\equiv f_{2}(G, M, C_{T}, T_{\beta }, I_{\gamma }), \nonumber \\ \frac{d C_{T}}{d t}= \, & {} \frac{a_{2}G}{k_{5}+T_{\beta }}-\mu _{1} C_{T} - \alpha _{4}\frac{G}{k_{3}+ G}C_{T} ~\equiv f_{3}(G, M, C_{T}, T_{\beta }, I_{\gamma }), \nonumber \\ \frac{d T_{\beta }}{d t}= \, & {} s_{1}+b_{1}G-\mu _{2} T_{\beta } ~\equiv f_{4}(G, M, C_{T}, T_{\beta }, I_{\gamma }), \nonumber \\ \frac{d I_{\gamma }}{d t}= \, & {} b_{2}C_{T}- \mu _{3}I_{\gamma } ~\equiv f_{5}(G, M, C_{T}, T_{\beta }, I_{\gamma }). \end{aligned}$$

The biologically feasible steady states of the temporal model (8) are the positive solutions of the following equations $f_{i}(G, M, C_{T}, T_{\beta }, I_{\gamma }) ~= 0,$ for $i = 1$ to 5. The model system (8) has three biologically relevant steady states, namely

(i) boundary steady state $E_{1}(0, 0, 0, T_{\beta }, 0)$, that is, $E_{1}(0, 0, 0, \frac{s_{1}}{\mu _{2}}, 0)$,
(ii) glioma cell free steady state ${\bar{E}}(0, {\bar{M}}, 0, {\overline{T}}_{\beta }, 0)$, that is, ${\bar{E}}(0, m_{1}, 0, \frac{s_{1}}{\mu _{2}}, 0)$, and
(iii) an interior equilibrium point $E^{*} (G^{*}, M^{*}, C_{T}^{*}, T_{\beta }^{*}, I_{\gamma }^{*}).$

The interior steady state $E^{*}$ is difficult to study explicitly. By using baseline set of model parameters stated in the Table 1, the model system (1)–(5) has only one positive interior fixed point $E^{*}$ is approximately given by $G^{*} = 875419,$ $M^{*} = 943092,$ $C_{T}^{*} = 303.397,$ $T_{\beta }^{*} = 9134.33,$ and $I_{\gamma }^{*} = 0.303397$.

**Table 1:** Description of the parameter values used for computer simulations.

Parameter	Description	Value/range	Units	Source
$r_{1}$	Growth rate of malignant gliomas	0.01–0.022	h^−1^	^16^
$G_{\max }$	Carrying capacity of glioma cells	$8.8265\times 10^{5}$	Cell	^16^
$e_{1}$	Michaelis-menten constant	$10^{4}$	pg	^18^
$\alpha _{1}$	Loss of macrophages due to glioma cells	1.5	pg h^−1^	^52^
$\alpha _{2}$	Loss of CD8+T cells due to glioma cells	0.12	pg h^−1^	^16^
${D_{G}}$	Diffusion rate of glioma cells	0.1 - 100	cm^2^ s^−1^	^25,45^
${D_{M}}$	Diffusion rate macrophages	5	cm^2^ s^−1^	^15^
${D_{C}}$	Diffusion rate of CD8+T cells	25	cm^2^ s^−1^	^15^
$k_{1}$	Half-saturation constant	$2.7 \times 10^{4}$	Cell	^52^
$r_{2}$	Proliferation rate of macrophages	0.3307	h^−1^	^16^
$M_{\max }$	Carrying capacity of macrophages	$10^{6}$	Cell	^16^
$a_{1}$	Maximum efficiency of IFN-$\gamma$	0.1163	Cell h^−1^	^16^
$k_{4}$	Half-saturation constant	$1.05 \times 10^{4}$	pg	^16^
$e_{2}$	Michaelis-menten constant	$10^{4}$	pg	^18^
$\alpha _{3}$	Anti-proliferative effect of macrophages	0.0194	h^−1^	^52^
$k_{2}$	Half saturation constant	$2.7 \times 10^{4}$	Cell	^16^
$a_{2}$	Antigenicity of gliomas	$0 - 0.5$	h^−1^ pg	^16^
$k_{5}$	Inhibitory constant	$2\times 10^{3}$	pg	^17^
$\mu _{1}$	Natural death rate of CD8+T cells	0.0074	h^−1^	^11^
$\alpha _{4}$	Anti-proliferative effect of CD8+T cells	0.1694	h^−1^	^17^
$k_{3}$	Half-saturation constant	$3.3445\times 10^{5}$	Cell	^16^
$s_{1}$	Source of TGF-$\beta$ in the CNS	$6.3305 \times 10^{4}$	pg h^−1^	^18^
$b_{1}$	Source rate of TGF-$\beta$	$5.75\times 10^{-6}$	pg cell^−1^ h^−1^	^16^
$\mu _{2}$	Degradation rate of TGF-$\beta$	6.931	h^−1^	^56^
$b_{2}$	Release rate of CD8+T cells	$1.02\times 10^{-4}$	pg cell^−1^ h^−1^	^16^
$\mu _{3}$	Natural decay rate of IFN-$\gamma$	0.102	h^−1^	^16^

In order to perform local stability analysis for the system (8), we evaluate the Jacobian matrix corresponding to each of the steady state $E(G, M, C_{T}, T_{\beta }, I_{\gamma })$ is given by

$$\begin{aligned} J_{E}= \left[ J(G, M, C_{T}, T_{\beta }, I_{\gamma }) \right] _{E} = \left( \begin{array}{ccccc} m_{11} &{} m_{12} &{} m_{13} &{} m_{14} &{} 0 \\ m_{21} &{} m_{22} &{} 0 &{} m_{24} &{} m_{25} \\ m_{31} &{} 0 &{} m_{33} &{} m_{34} &{} 0 \\ m_{41} &{} 0 &{} 0 &{} m_{44} &{} 0 \\ 0 &{} 0 &{} m_{53} &{} 0 &{} m_{55} \\ \end{array} \right) , \end{aligned}$$

where

$$\begin{aligned} \begin{array}{lll} m_{11}=r_{1}\left( 1-\frac{2G}{G_{\max }}\right) -\frac{k_{1} (\alpha _{1}M+\alpha _{2}C_{T})}{(T_{\beta }+e_{1})(G+k_{1})^{2}}, &{} m_{12}=-\frac{\alpha _{1}G}{(T_{\beta }+e_{1})(G+k_{1})}, &{} m_{13}=-\frac{\alpha _{2}G}{(T_{\beta }+e_{1})(G+k_{1})}, \\ m_{22}=r_{2}(1-\frac{2M}{M_{\max }})-\frac{\alpha _{3}G}{G+k_{2}},&{} m_{14}=\frac{G(\alpha _{1}M+\alpha _{2}C_{T})}{(T_{\beta }+e_{1})^{2}(G+k_{1})},&{} m_{21}=-\frac{\alpha _{3}k_{2}M}{(G+k_{2})^{2}}, \\ m_{24}=-\frac{a_{1}I_{\gamma }}{(k_{4}+I_{\gamma })(T_{\beta }+e_{2})^{2}},&{} m_{25}=\frac{a_{1}k_{4}}{(k_{4}+I_{\gamma })^{2}(T_{\beta }+e_{2})},&{} m_{31}=\frac{a_{2}}{k_{5}+T_{\beta }}-\frac{\alpha _{4}k_{3}C_{T}}{(G+k_{3})^{2}}, \\ m_{33}=-\mu _{1}-\frac{\alpha _{4}G}{G+k_{3}},&{} m_{34}=-\frac{a_{2}G}{(k_{5}+T_{\beta })^{2}},&{} m_{41}=b_{1}, \\ m_{44}=-\mu _{2},&{} m_{53}=b_{2},&{}m_{55}=-\mu _{3}. \\ \end{array} \end{aligned}$$

At the boundary steady state $E_{1}(0, 0, 0, \frac{s_{1}}{\mu _{2}}, 0)$, the eigenvalues of the Jacobian matrix $J_{E_{1}}$ are $\lambda _{1}=r_{1}$, $\lambda _{2}=r_{2}$, $\lambda _{3}=-\mu _{1}$, $\lambda _{4}=-\mu _{2}$, and $\lambda _{5}=-\mu _{3}$. From the eigenvalues it is obvious that the boundary steady state $E_{1}$ is hyperbolic saddle type with two dimensional unstable manifold and three dimensional stable manifold. From the biomedical view point, the boundary equilibrium point $E_{1}$ has limited interest in the glioma-immune interactive dynamics as only the immuno-suppressive cytokine TGF-$\beta$ present.

At the cancer free or glioma-free equilibrium point ${\bar{E}}(0, M_{\max }, 0, \frac{s_{1}}{\mu _{2}}, 0)$ of the system (8) has the eigenvalues of $J_{{\bar{E}}}$ are given by $\lambda _{1}=r_{1}-\frac{\alpha _{1}M_{\max }\mu _{2}}{k_{1}(s_{1}+e_{1}\mu _{2})}$, $\lambda _{2}=-r_{2}$, $\lambda _{3}=-\mu _{1}$, $\lambda _{4}=-\mu _{2}$ and $\lambda _{5}=-\mu _{3}.$ If $\alpha _{1} < \frac{r_{1}k_{1}(s_{1}+e_{1}\mu _{2})}{\mu _{2}M_{\max }},$ then $Re(\lambda _{1}) < 0$ hence, ${\bar{E}}$ is locally asymptotically stable. If $\alpha _{1} > \frac{r_{1}k_{1}(s_{1}+e_{1}\mu _{2})}{\mu _{2}M_{\max }},$ then $Re(\lambda _{1}) > 0$ hence, ${\bar{E}}$ is saddle type. If $\alpha _{1} = \frac{r_{1}k_{1}(s_{1}+e_{1}\mu _{2})}{\mu _{2}M_{\max }},$ then $Re(\lambda _{1}) = 0$ gives zero eigenvalue of $J_{{\bar{E}}}$ and we cannot conclude about the stability of ${\bar{E}}$. The preceding argument indicates that the proliferation rate of malignant gliomas $r_{1}$ as a critical parameter, which characterizes the stability of the glioma-free equilibrium point $E_{2}$. From the biomedical view point the glioma-free singular point ${\bar{E}}$ has an impact as it gives an idea under what circumstances the patients’ will be free from malignant glioma cell population.

Now, we shall investigate the asymptotic stability of an interior steady state $E^{*}(G^{*}, M^{*},$ $C_{T}^{*}, T_{\beta }^{*}, I_{\gamma }^{*})$ for the temporal system (8). Therefore, the characteristic equation of the Jacobian matrix $J_{E^{*}}$ is given by

$$\begin{aligned} \lambda ^{5}+\rho _{1}\lambda ^{4}+\rho _{2}\lambda ^{3}+\rho _{3}\lambda ^{2} +\rho _{4}\lambda +\rho _{5}=0, \end{aligned}$$

where

$$\begin{aligned} \rho _{1}= \, & {} - m_{11} -m_{22} -m_{33}-m_{44}-m_{55}, \\ \rho _{2}= \, & {} -m_{12} m_{21}+m_{11} m_{22} -m_{13} m_{31} +m_{11} m_{33}+m_{22} m_{33} -m_{14} m_{41} +m_{11} m_{44}+m_{22} m_{44} \\&+ m_{33} m_{44} +m_{11}m_{55} +m_{22} m_{55}+m_{33} m_{55}+m_{44} m_{55}, \\ \rho _{3}= \, & {} m_{13} m_{22} m_{31} +m_{12} m_{21} m_{33} -m_{11} m_{22} m_{33} +m_{14} m_{22} m_{41} -m_{12} m_{24} m_{41}+m_{14} m_{33} m_{41} \\&- m_{13} m_{34} m_{41} + m_{12} m_{21} m_{44}-m_{11} m_{22} m_{44} +m_{13} m_{31} m_{44}-m_{11} m_{33} m_{44}-m_{22} m_{33} m_{44} \\&+ m_{12} m_{21} m_{55} - m_{11} m_{22} m_{55}+m_{13} m_{31} m_{55}-m_{11} m_{33} m_{55}-m_{22} m_{33} m_{55}+m_{14} m_{41} m_{55} \\&- m_{11} m_{44} m_{55}-m_{22} m_{44} m_{55}-m_{33} m_{44} m_{55}, \\ \rho _{4}= \, & {} -m_{14} m_{22} m_{33} m_{41} +m_{12} m_{24} m_{33} m_{41}+m_{13} m_{22} m_{34} m_{41} -m_{13} m_{22} m_{31} m_{44}-m_{12} m_{21} m_{33} m_{44} \\&+ m_{11} m_{22} m_{33} m_{44}-m_{12} m_{25} m_{31} m_{53}-m_{13} m_{22} m_{31} m_{55} -m_{12} m_{21} m_{33} m_{55} +m_{11} m_{22} m_{33} m_{55} \\&- m_{14} m_{22} m_{41} m_{55} +m_{12} m_{24} m_{41} m_{55}-m_{14} m_{33} m_{41} m_{55} +m_{13} m_{34} m_{41} m_{55}-m_{12} m_{21} m_{44} m_{55} \\&+ m_{11} m_{22} m_{44} m_{55} -m_{13} m_{31} m_{44} m_{55}+m_{11} m_{33} m_{44} m_{55}+m_{22} m_{33} m_{44} m_{55}, \\ \rho _{5}= \, & {} -m_{12} m_{25} m_{34} m_{41} m_{53}+m_{12} m_{25} m_{31} m_{44} m_{53} + m_{14} m_{22} m_{33} m_{41} m_{55} - m_{12} m_{24} m_{33} m_{41} m_{55} \\&- m_{13} m_{22} m_{34} m_{41} m_{55} + m_{13} m_{22} m_{31} m_{44} m_{55}+m_{12} m_{21} m_{33} m_{44} m_{55} - m_{11} m_{22} m_{33} m_{44} m_{55}. \\ \end{aligned}$$

Due to the well-known Routh–Hurwitz criterion, the interior equilibrium point $E^{*}$ is asymptotically stable if the following conditions: $\rho _{j} > 0$, (for $j = 1$, 2, 3, 4, 5), $\rho _{1} \rho _{2} - \rho _{3} > 0$, $\rho _{1} \rho _{2} \rho _{3} > \rho _{1}^{2} \rho _{4} + \rho _{3}^{2}$ and $(\rho _{3} \rho _{4}- \rho _{2} \rho _{5})(\rho _{1}\rho _{2}-\rho _{3})>(\rho _{1}\rho _{4}-\rho _{5})^{2}$ are satisfied. For the parameter values defined in the Table 1, the system has a unique biologically relevant, nonnegative interior steady state $E^{*}$, and the characteristics equation becomes

$$\begin{aligned} \lambda ^{5} + 7.48429 \lambda ^{4} + 3.92565 \lambda ^{3} + 0.634199 \lambda ^{2} + 0.0344357 \lambda + 0.000281075 = 0, \end{aligned}$$

which satisfies all the conditions for the Routh–Hurwitz criteria ($\rho _{1} \rho _{2} - \rho _{3} = 28.7465 > 0$, $\rho _{1} \rho _{2} \rho _{3} - \rho _{1}^{2} \rho _{4} - \rho _{3}^{2} = 16.3021 > 0$, $(\rho _{3} \rho _{4}- \rho _{2} \rho _{5})(\rho _{1}\rho _{2}-\rho _{3}) - (\rho _{1}\rho _{4}-\rho _{5})^{2} = 0.5298 > 0$) and the associated eigenvalues are $- 6.931,$ $- 0.311883,$ $- 0.129568,$ $- 0.102002$ and $- 0.00983845$. All the eigenvalues are negative and have negative real parts, therefore the temporal system (8) is locally asymptotically stable. From the medical point of view, the interior steady state $E^{*}$ has a great impact as it gives us an idea under what situation all the cell populations including glioma cell population persists. For the parameter set in the Table 1, our model system has a unique interior steady state and it is locally asymptotically stable. Whatever initial conditions may be the system will be locally asymptotically stable and goes to an interior singular point. Thus, we leave the global stability analysis.

Now, we shall explore the dynamics of diffusion-driven instability with respect to the interior steady state, that is, the spatially homogeneous steady-state solution $E^{*}(G^{*},$ $M^{*},$ $C_{T}^{*},$ $T_{\beta }^{*}, I_{\gamma }^{*})$ for the reaction-diffusion system (1)–(5). Clearly, the interior steady state $E^{*}$ for the temporal model (8) is a spatially homogeneous steady-state for the reaction-diffusion system (1)–(5). In this section, we assume that the homogeneous interior steady state $E^{*}$ is asymptotically stable for the temporal system, which indicate that the spatially homogeneous steady state is locally stable with reference to spatially homogeneous perturbations. Diffusivity is often referred as a stabilizing process, yet it is well-known that, in a system of interacting populations, diffusion can make a spatially homogeneous equilibrium linearly unstable with respect to heterogeneous perturbation^14,35,58^. The diffusion-induced instability happen when a temporally stable steady-state become destabilized due to the diffusive nature of the interactive populations^14^.

To study the conditions for diffusion-driven destabilization of the temporal system, one should investigate how small inhomogeneous perturbations of the homogeneous equilibrium point acts in the large-time limit. To notice the scenario, we choose the following two dimensional Fourier modes as the perturbation function as

9 $$\begin{aligned} G(x, y, t)= \, & {} G^{*} + \xi \exp ( (l_{x}x + l_{y} (y))i + \lambda _{l} t) , \nonumber \\ M(x, y, t)= \, & {} M^{*} + \sigma \exp ( (l_{x}x + l_{y} (y))i + \lambda _{l} t) , \nonumber \\ C_{T}(x, y, t)= \, & {} C_{T}^{*} + \epsilon \exp ( (l_{x}x + l_{y} (y))i + \lambda _{l} t) , \nonumber \\ T_{\beta }(x, y, t)= \, & {} T_{\beta }^{*} + \eta \exp ( (l_{x}x + l_{y} (y))i + \lambda _{l} t) , \nonumber \\ I_{\gamma }(x, y, t)= \, & {} I_{\gamma }^{*} + \rho \exp ( (l_{x}x + l_{y} (y))i + \lambda _{l} t) , \end{aligned}$$

where $\xi$, $\sigma$, $\epsilon$, $\eta$ and $\rho$ are very small and nonnegative constants, $\lambda _{l}$ is the activation rate of perturbation at time t, and $l = \left( l_{x}, l_{y} \right)$ is the wave number of the solutions. In the spatiotemporal system, the magnitude of $\lambda _{l}$ depends on the sum of the square of the wave numbers $l^{2} = l_{x}^{2} + l_{y}^{2}$^58^. As a result, the eigenvalues are influenced by wave numbers. It makes more clear that some of the Fourier modes will disappear in the large-time limit whereas others will amplify. For notational clarity, we consider $\lambda _{l}$ as a rotational symmetric function on $l_{x}l_{y}-$plane and substituting $l^{2} = l_{x}^{2} + l_{y}^{2}$, we obtain the two-dimensional case from one-dimensional derivation. On substitution of (9) into the model system (1)–(5), we linearize the system around interior singular point $E^{*}$, we get

10 $$\begin{aligned} {\underline{w}}_{t} = \mathbf {J_{E}} + {\mathbf {M}} \nabla ^{2} {\underline{w}}, \end{aligned}$$

where $w = (G, M, C_{T}, T_{\beta }, I_{\gamma })^{T}$, $\mathbf {J_{E}}$ is the variational matrix of the spatially homogeneous model system (1)–(5) and ${\mathbf {M}}$ is the corresponding matrix for diffusion coefficients;

$$\begin{aligned} {\mathbf {M}} = \left( \begin{array}{ccccc} D_{G} &{} 0 &{} 0 &{} 0 &{} 0 \\ 0 &{} D_{M} &{} 0 &{} 0 &{} 0 \\ 0 &{} 0 &{} D_{C} &{} 0 &{} 0 \\ 0 &{} 0 &{} 0 &{} D_{T} &{} 0 \\ 0 &{} 0 &{} 0 &{} 0 &{} D_{I} \\ \end{array} \right) . \end{aligned}$$

After the substitution of (9) into the system of equations (1)–(5), the solutions of the linearized system are proportional to $w = \exp ( (l_{x}x + l_{y} (y))i + \lambda _{l} t)$ and using standard linear stability analysis^35^ the system of partial differential equations (10) leads to the following version of the spatial model system as

11 $$\begin{aligned} \lambda {\underline{w}} = \mathbf {J_{E}} w - {\mathbf {M}} l^{2} {\underline{w}}, \end{aligned}$$

where *l* is spatial wave number defined as before.

The eigenvalues $(\lambda )$ of the linearized model system (11) are found by the solutions of the following characteristics equation as follows

$$\begin{aligned} \left| \left( \mathbf {J_{E}} - {\mathbf {M}} l^{2} \right) - \lambda {\mathbf {I}} \right| = \left[ \left( \mathbf {J_{E}} - {\mathbf {M}} l^{2} \right) - \lambda {\mathbf {I}} \right] \left[ \begin{array}{c} G \\ M \\ C_{T} \\ T_{\beta } \\ I_{\gamma } \end{array} \right] = 0. \end{aligned}$$

Explicitly, we can write that

$$\begin{aligned} \left[ \begin{array}{ccccc} e_{11} - \lambda &{} m_{12} &{} m_{13} &{} m_{14} &{} 0 \\ m_{21} &{} e_{22} - \lambda &{} 0 &{} m_{24} &{} m_{25} \\ m_{31} &{} 0 &{} e_{33} - \lambda &{} m_{34} &{} 0 \\ m_{41} &{} 0 &{} 0 &{} e_{44} - \lambda &{} 0 \\ 0 &{} 0 &{} m_{53} &{} 0 &{} e_{55} - \lambda \\ \end{array} \right] \left[ \begin{array}{c} G \\ M \\ C_{T} \\ T_{\beta } \\ I_{\gamma } \end{array} \right] = \left[ \begin{array}{c} 0 \\ 0 \\ 0 \\ 0 \\ 0 \end{array} \right] . \end{aligned}$$

with

$$\begin{aligned} \left\{ \begin{array}{ll} e_{11} = m_{11} - D_{G} l^{2} \\ e_{22} = m_{22} - D_{M} l^{2} \\ e_{33} = m_{33} - D_{C} l^{2} \\ e_{44} = m_{44} - D_{T} l^{2} \\ e_{55} = m_{11} - D_{G} l^{2}, \end{array} \right. \end{aligned}$$

which is a fifth degree polynomial in $\lambda$ and *l*. For the parameters value defined in the Table 1, we solve the characteristic equation in the numerical section. For that, we plot the dispersion curves for the characteristic polynomial to investigate how the value of the real part of the eigenvalues $(Re(\lambda ))$ varies with respect to *l*.

## Sensitivity analysis

The model for glioma-immune interplays given in equations (1)–(5) is investigated so that the system parameters are taken from different sources including existing literatures, experimental works and *in vivo* data due to their wide variability. Thus, a sensitivity analysis is conducted by using Partial Rank Correlation Coefficient (PRCC) technique to determine how the glioma-immune model output is affected by changes in a specific parameter irrespective the uncertainty over the rest of the parameters. In our model, we have 23 parameters for which we varied all the model parameters simultaneously. PRCC quantifies the relationship between a state equation of interest and each model parameter. As a result PRCC help to find the important parameters, which contribute most to the system variability. For our model simulation, we consider the PRCC values between − 1 and + 1.

**Figure 2:**
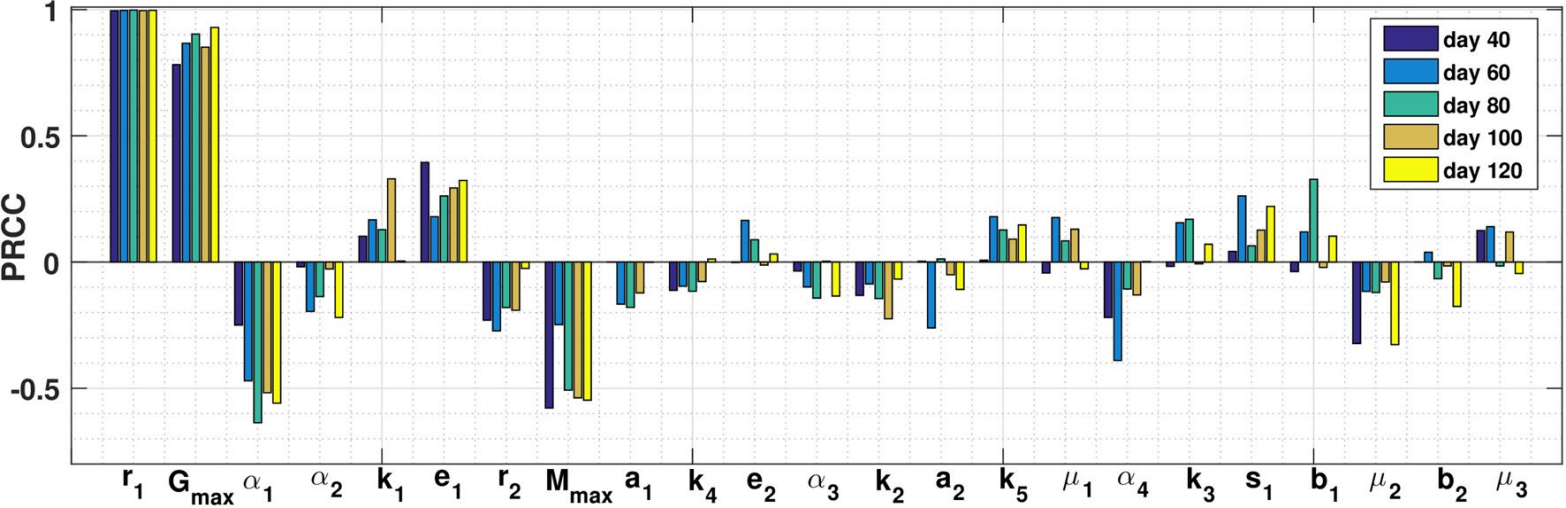
PRCC results indicating sensitivity indices for the system parameters in absence of diffusion with glioma cell population chosen as a baseline PRCC variable at five different time points with $p < 0.0001$.

Following the method developed by Marino et al.^59^, we performed Latin hypercube sampling and generated 2500 samples to compute the PRCC and p-values with reference to the glioma cells for five different time points in order to determine which parameters consistently influence the model output. The indexes are computed at the following time points 40, 60, 80, 100 and 120 days prior to equilibrium state. Figure 2 represents the PRCC results, which indicates that the parameters for glioma proliferation rate, $r_1$; glioma cells carrying capacity, $G_{max}$; deactivation rate of glioma cells due to macrophages, $\alpha _{1}$ and the carrying capacity of macrophages, $M_{\max }$ account for most uncertainty of the malignant glioma cell population. Figure 2 exhibits the PRCC results, which indicate that the malignant gliomas over time is mainly influenced due to variation of the parameters $r_1$, $G_{max}$, $\alpha _{1}$ and $M_{\max }$.

From the PRCC Fig. 2 it can be observed that the proliferation rate of glioma cells $r_1$ and the carrying capacity of glioma cell population $G_{\max }$ are highly positively correlated with respect to glioma cells. It is interesting to observe that the rate $\alpha _1$, by which the glioma cell population reduces due to interaction with macrophages population, is highly negatively correlated, that is, when the blockade of the macrophages is increased, the concentration of glioma cells is decreased. It can also be observed that the carrying capacity of macrophages $M_{\max }$ is highly negatively correlated.

## Spatiotemporal dynamics

In this section, extensive numerical simulations are carried out for the glioma-immune interaction model (1)–(5) in both one and two-dimensional domains. In order to compute the numerical simulations of our model, we use finite difference approximations to perform a spatial discretization of the model equations. Now, we shall discuss the numerical scheme that has been used in our numerical solutions.

### Numerical algorithm

The nonlinear reaction-diffusion system (1)-(5) was solved numerically by using MATLAB. Numerical integrations of (1)–(5) are performed by transforming the infinite dimensional continuous system to a finite dimensional form using discritization with respect to time and space. The forward Euler method is utilized for non-diffusive part and standard five-point finite difference method is utilized for diffusion part. We performed computer illustrations over a $1000 \times 1000$ lattice sites with spacing between two neighboring lattice points are considered as $\Delta x = \Delta y = 1$ with $\Delta t = 0.002$. To avoid numerical stiffness, we performed the simulations qualitatively for smaller values of the step sizes. It is important to note that the patterns studied in this manuscript are not dependent on the time-step $\Delta t$. For the one-dimensional case, the wave number *l* is scalar and the corresponding single pattern appears, but for the case of two-dimensional system, the corresponding wave number $(l_{x}, l_{y})$ is two-dimensional, where $l = \sqrt{l_{x}^{2} + l_{y}^{2}}$ and complex patterns appear. The detailed numerical scheme and algorithm are given in the Supplementary Material.

Zero-flux boundary conditions has been used at the boundary of square domain to perform the numerical simulations. We have taken same initial conditions for each numerical simulation and the initial density of glioma cell population, macrophages, CD8+ T cells, TGF-β and IFN-γ are chosen in such a way that they are located inside the domain. The choice of initial condition indicates small inhomogeneous spatial perturbation from homogeneous equilibrium state. In case of one-dimensional system, the initial distribution of the cell populations are taken as

$$\begin{aligned} \left\{ \begin{array}{ll} G(x, 0)= C_{T}(x, 0)= T_{\beta }(x, 0) = I_{\gamma }(x, 0) = \epsilon (x-300)^{2}< 900 , &{} x \in \Omega , \epsilon> 0 \\ M(x, 0)= \eta (x+300)^{2} < 900 , &{} x \in \Omega , \eta > 0, \end{array} \right. \end{aligned}$$

and for the case of two-dimensional system, the initial distribution of the cell populations are taken as

$$\begin{aligned} \left\{ \begin{array}{lll} G(x, y, 0)= C_{T}(x, y, 0) = \epsilon ((x-300)^{2} + (y-300)^{2})< 900 , &{} (x, y) \in \Omega , \epsilon> 0, \\ T_{\beta }(x, y, 0) = I_{\gamma }(x, y, 0) = \epsilon ((x-300)^{2} + (y-300)^{2})< 900 , &{} (x, y) \in \Omega , \epsilon> 0, \\ M(x, y, 0)= \eta ((x+300)^{2} + (y+300)^{2}) < 900 , &{} (x, y) \in \Omega , \eta > 0. \end{array} \right. \end{aligned}$$

### Results

**Figure 3:**
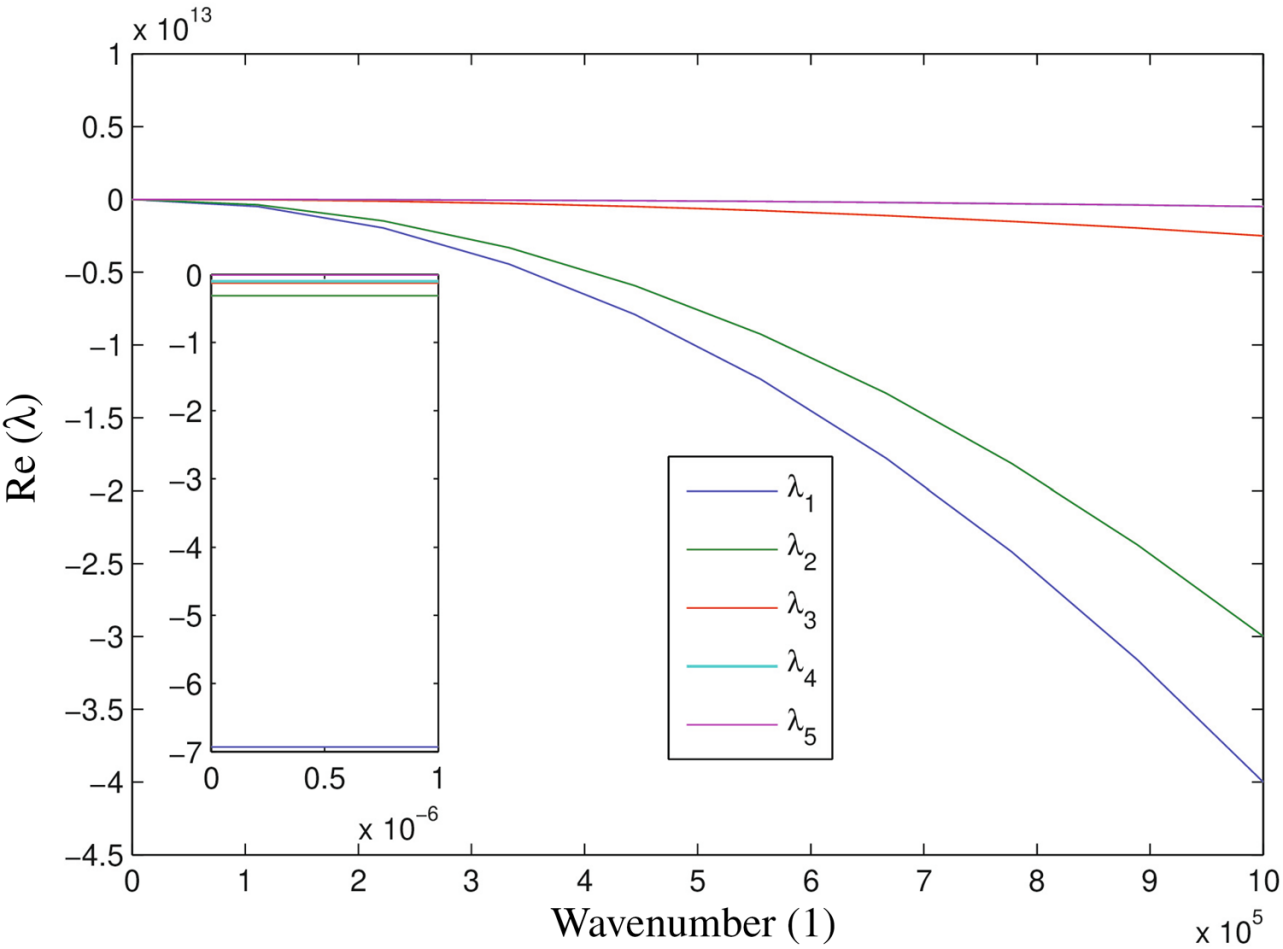
Numerically simulated dispersion curves for the model system (1)–(5) of wavenumber(l) (x-axis) against the real part of $\lambda$ (y-aixs) with $D_{G} = 55,$ $D_{M} = 5,$ $D_{T} = 400,$ $D_{C} = D_{I} = 25$ and rest of the parameters are defined in Table 1. Five different curves are designate for different eigenvalues. The dispersion curve is computed by obtaining the real part of eigenvalues for the linearized system by varying the wavenumber(l) from zero in increments of 0.001.

We have plotted the dispersion curve in the Fig. 3, which clearly shows that after inhomogeneous perturbation the spatiotemporal dynamics remain stable, that is, $Re(\lambda ) < 0$. Thus, there is no possibility of occurring the Turing pattern which is biologically relevant in glioma-immune interactive dynamics. We computed the eigenvalues of the corresponding characteristic equation for the range of $l \in [0, 10^{6}]$,

$$\begin{aligned}&~ \lambda ^{5} + \lambda ^{4} [73.5 l^{2} + 7.48429] + \lambda ^{3}[ 1447.75 l^{4} + 269.311 l^{2} + 3.92565] \\&\quad + \, \lambda ^{2}[ 4393.13 l^{6} + 1379.99 l^{4} + 109.013 l^{2} + 0.634199] + \lambda [3343.75 l^{8}+ 1931.52 l^{6} \\&\quad + \, 292.795 l^{4} + 10.4906 l^{2} + 0.034457 ] + (750 l^{10} + 653.957 l^{8} + 126.786 l^{6} + 6.82954 l^{4} \\&\quad + \, 0.104449 l^{2} + 0.000281075) = 0. \end{aligned}$$

It is important to note that for a larger interval of *l*, the largest real part of the eigenvalue is always negative which indicates that the model system (1)–(5) is always stable.

The biological motivation of this study is to investigate the heterogeneous nature of glioma cell population and immune components namely, macrophages and activated CD8+T cells. The two-dimensional system provides more realistic phenomenon with much complexity than one-dimensional system which is the main aim to investigate the solutions of the two-dimensional system.

**Figure 4:**
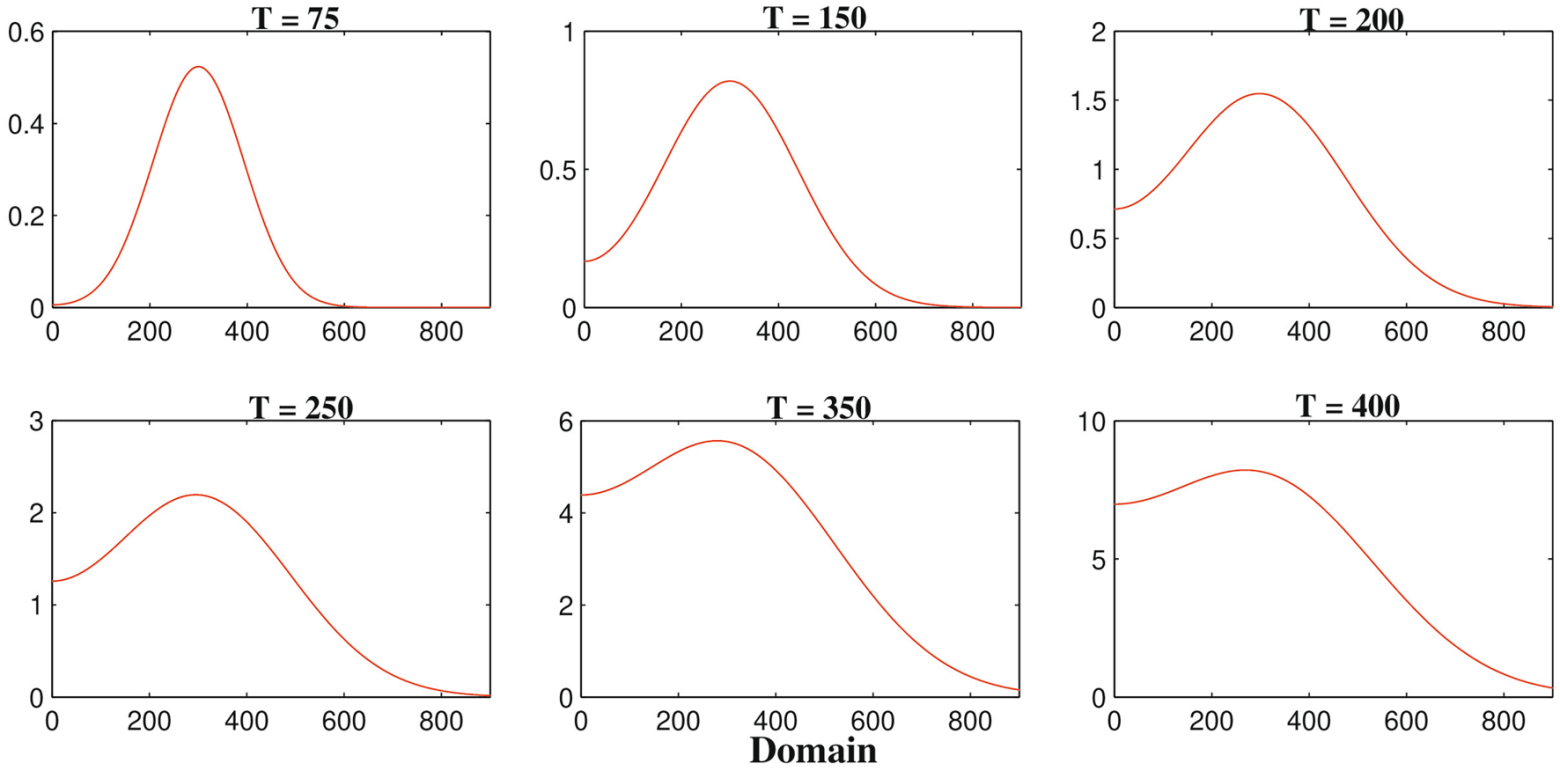
The one-dimensional numerical simulations describe the dynamics of glioma cells density *G*(*t*, *x*, *y*) at times corresponding to 75, 150, 200, 250, 350 and 400 h respectively. The simulation shows the migration of glioma cells density through the domain for an increasing value of time. Parameter values: $D_{G} = 55$, $D_{M} = 5$, $D_{T} = 400$, $D_{C} = D_{I} = 25$ and the other parameter values and initial conditions are presented in the Table 1.

**Figure 5:**
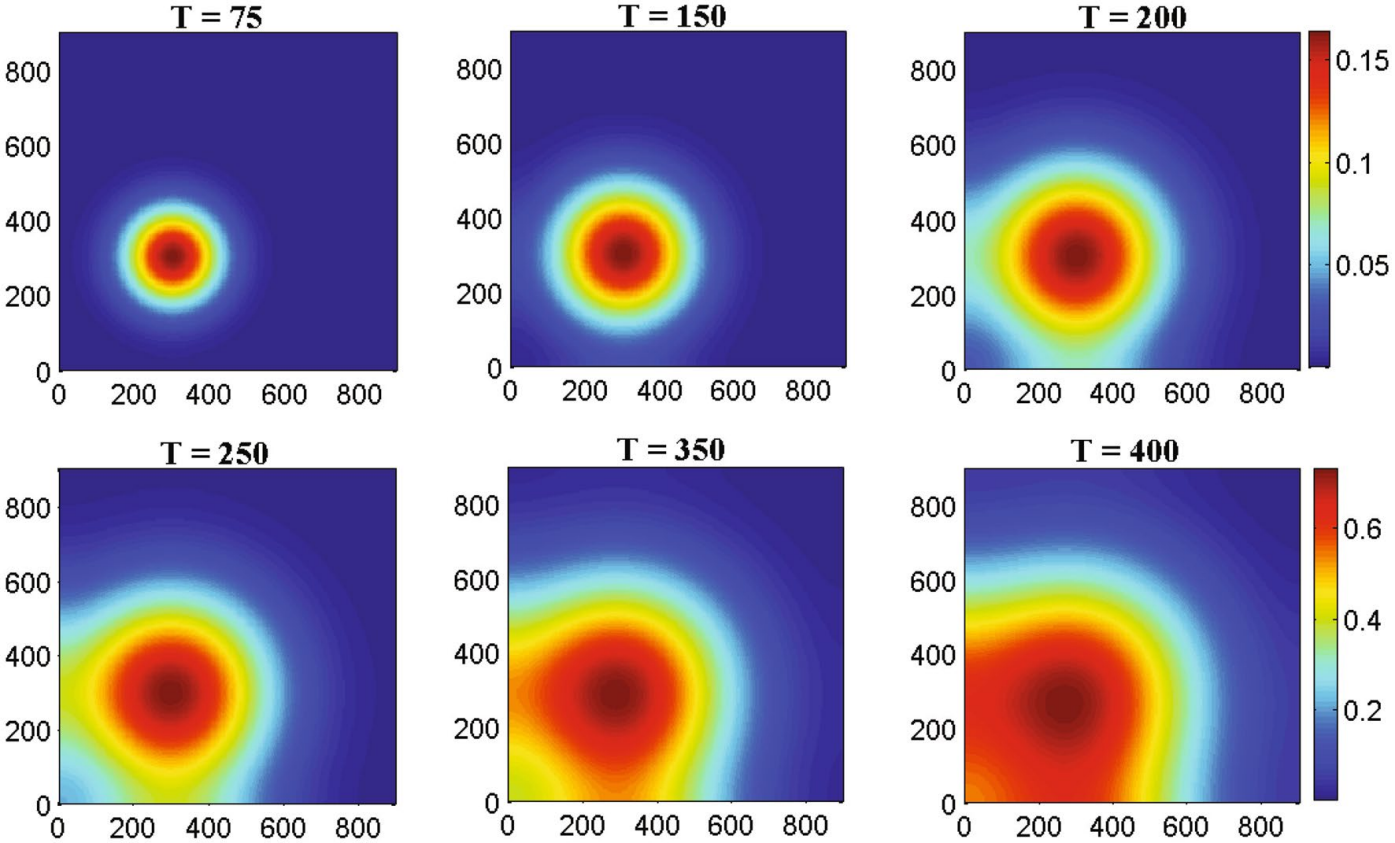
The two-dimensional numerical simulations describe the dynamics of glioma cells density *G*(*t*, *x*, *y*) at times corresponding to 75, 150, 200, 250, 350 and 400 h respectively. The simulation demonstrates the spatial distribution of glioma cells density through the domain for an increasing value of time. Parameter values: $D_{G} = 55$, $D_{M} = 5$, $D_{T} = 400$, $D_{C} = D_{I} = 25$ and the other parameter values and initial conditions are presented in the Table 1.

Figure 4 represents the result of numerical simulations of the density of malignant gliomas in one-dimensional domain whereas the corresponding two-dimensional scenarios are represented in the Fig. 5. Figure 4 shows that the one-dimensional heterogeneous spatial distribution of glioma density within brain tissue corresponding to times 75, 150, 200, 250, 350 and 400 h respectively. Initially, the glioma cells migrate a very small distance into the domain and as time evolves by $T =$ 400 h ($\sim$17 days) glioma cells migrate almost all over the domain. Figure 5 highlights different spread or migration pattern of gliomas at different times. In Fig. 5, initially at $T =$ 75 h ($\sim$3 days) the gliomas spread in a small mass into the domain. At, $T =$ 200 h ($\sim$8 days) glioma cells are spread or migrated almost one-fifth of the domain due to its aggressive nature. By the time $T =$ 350 h ($\sim$15 days) the initial cluster of glioma cells reaches almost in the left-hand boundary. At, $T =$ 400 h ($\sim$17 days) the initial cluster of glioma cell population reaches the left-hand boundary. In this connection, we assume that the left-hand boundary domain represents a zone of solid/rigid tissue or bone that the glioma cell populations are unable to infiltrate/penetrate/overcome (due to zero-flux boundary conditions) and hence they begin to move in the opposite direction, driven mainly by the macrophages and other immune components like activated cytotoxic T-lymphocytes, IFN-$\gamma$ and extracellular matrix components gradient.

**Figure 6:**
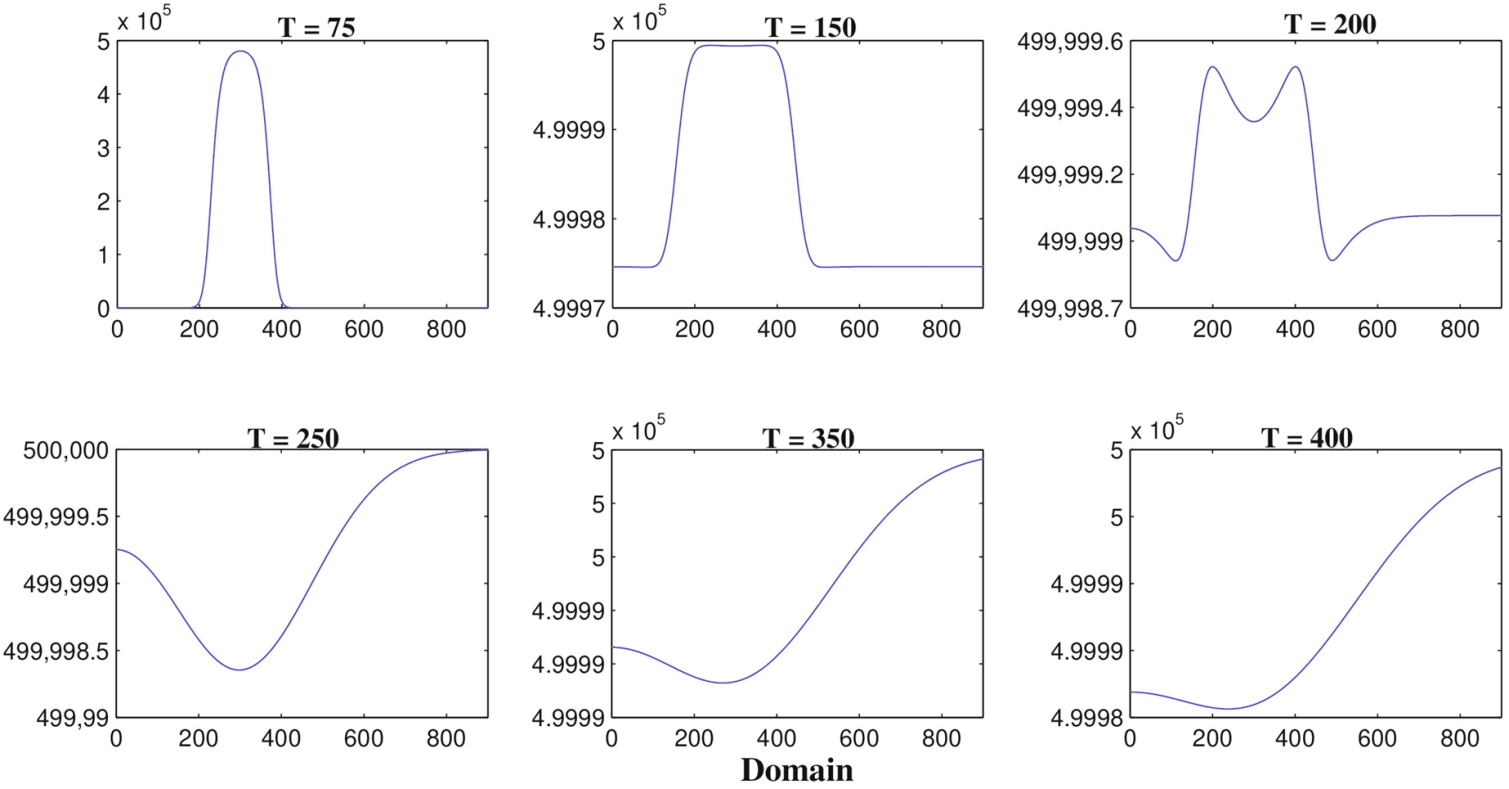
The one-dimensional numerical simulations describe the dynamics of macrophages density *M*(*t*, *x*, *y*) at times corresponding to 75, 150, 200, 250, 350 and 400 h respectively. The simulation shows the migration of macrophages density through the domain for an increasing value of time. Parameter values: $D_{G} = 55$, $D_{M} = 5$, $D_{T} = 400$, $D_{C} = D_{I} = 25$ and the other parameter values and initial conditions are presented in the Table 1.

**Figure 7:**
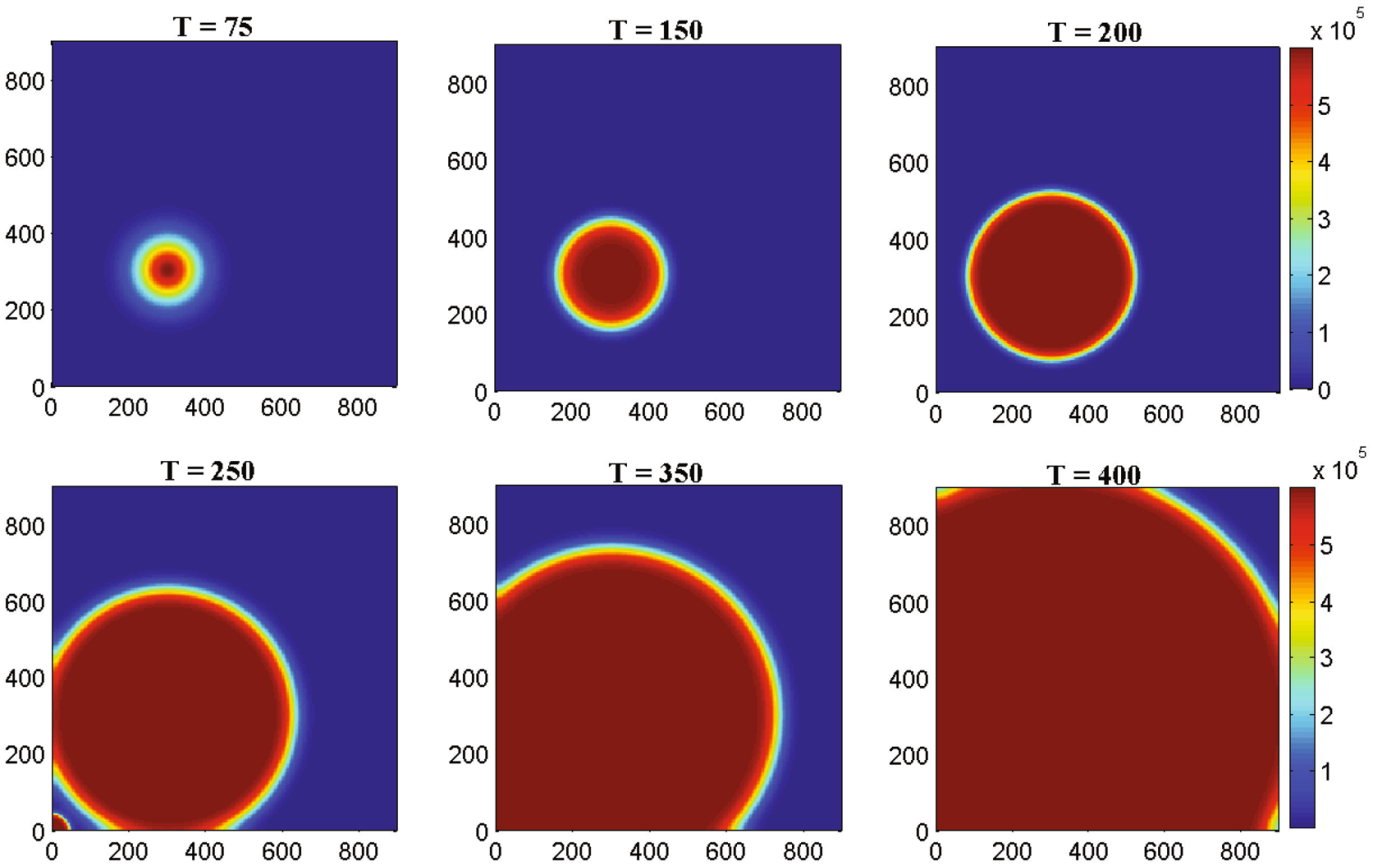
The two-dimensional numerical simulations describe the dynamics of macrophages density *M*(*t*, *x*, *y*) at times corresponding to 75, 150, 200, 250, 350 and 400 h respectively. The simulation demonstrates the spatial distribution of macrophages density through the domain for an increasing value of time. Parameter values: $D_{G} = 55$, $D_{M} = 5$, $D_{T} = 400$, $D_{C} = D_{I} = 25$ and the other parameter values and initial conditions are presented in the Table 1.

Figure 6 describes the results of one-dimensional numerical simulations of macrophages whereas the analogous two-dimensional shaded plots are shown in the Figure 7 under different scenarios of time in hours. Figure 6 shows that the spatiotemporal dynamics of macrophages population within the brain tissue corresponding to times 75, 150, 200, 250, 350 and 400 h respectively. Initially, at $T =$ 75 h ($\sim$3 days) the macrophages are small in size and it migrates little distance into the domain. By $T =$ 250 h ($\sim$11 days) macrophages have moved almost half of the domain maybe due to pseudopodia-mediated movement. From Figs. 6 and 7 it is clear that the migration rate of macrophages is faster than malignant glioma cells which has been observed in the Figs. 4 and 5, respectively. Hence, as the time increased at $T =$ 400 h ($\sim$17 days) macrophages population continue to move towards the entire domain. In the Fig. 7, we described the behavior of macrophages in two-dimensional shaded plot to see the more clear scenarios of how the macrophages migrate so quickly throughout the entire square domain than malignant gliomas. To illustrate the migration of macrophages or the wave of macrophages, we generated the snapshots of two-dimensional domains with times 75, 150, 200, 250, 350 and 400 h respectively. The distribution of macrophages at $T =$ 75 h ($\sim$3 days), in Fig. 7, describes that a small cluster of macrophages is built up but, that is spread in a large amount for $T =$ 150 h ($\sim$6 days) and $T =$ 200 h ($\sim$8 days) and so on, maybe due to increased haptotactic migration. In Fig. 7, at $T =$ 250 h ($\sim$11 days) the macrophages spread almost half of the domain and it is spread more faster than glioma cell population compared to Fig. 5 at $T =$ 250 h ($\sim$11 days). At, $T =$ 400 h ($\sim$17 days) the macrophages spread almost the entire rectangular domain (see the Fig. 7) and reaches the right-hand boundary of the domain, which may be explained as the right-hand boundary described as a zone of rigid tissue or bone that the macrophages are unable to overcome and they begin to move in the opposite direction to their initial direction, mainly by chemotaxis^53^ and pseudopodia-mediated migration.

**Figure 8:**
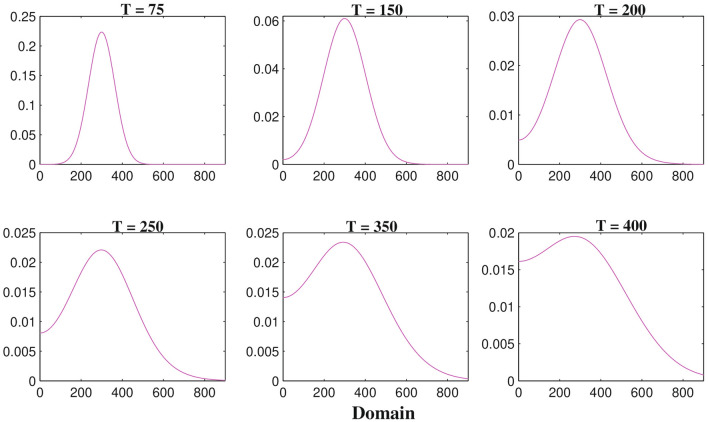
The one-dimensional numerical simulations describe the dynamics of cytotoxic T-lymphocytes density $C_{T}(t, x, y)$ at times corresponding to 75, 150, 200, 250, 350 and 400 h respectively. The simulation shows the migration of cytotoxic T-lymphocytes density through the domain for an increasing value of time. Parameter values: $D_{G} = 55$, $D_{M} = 5$, $D_{T} = 400$, $D_{C} = D_{I} = 25$ and the other parameter values and initial conditions are presented in the Table 1.

**Figure 9:**
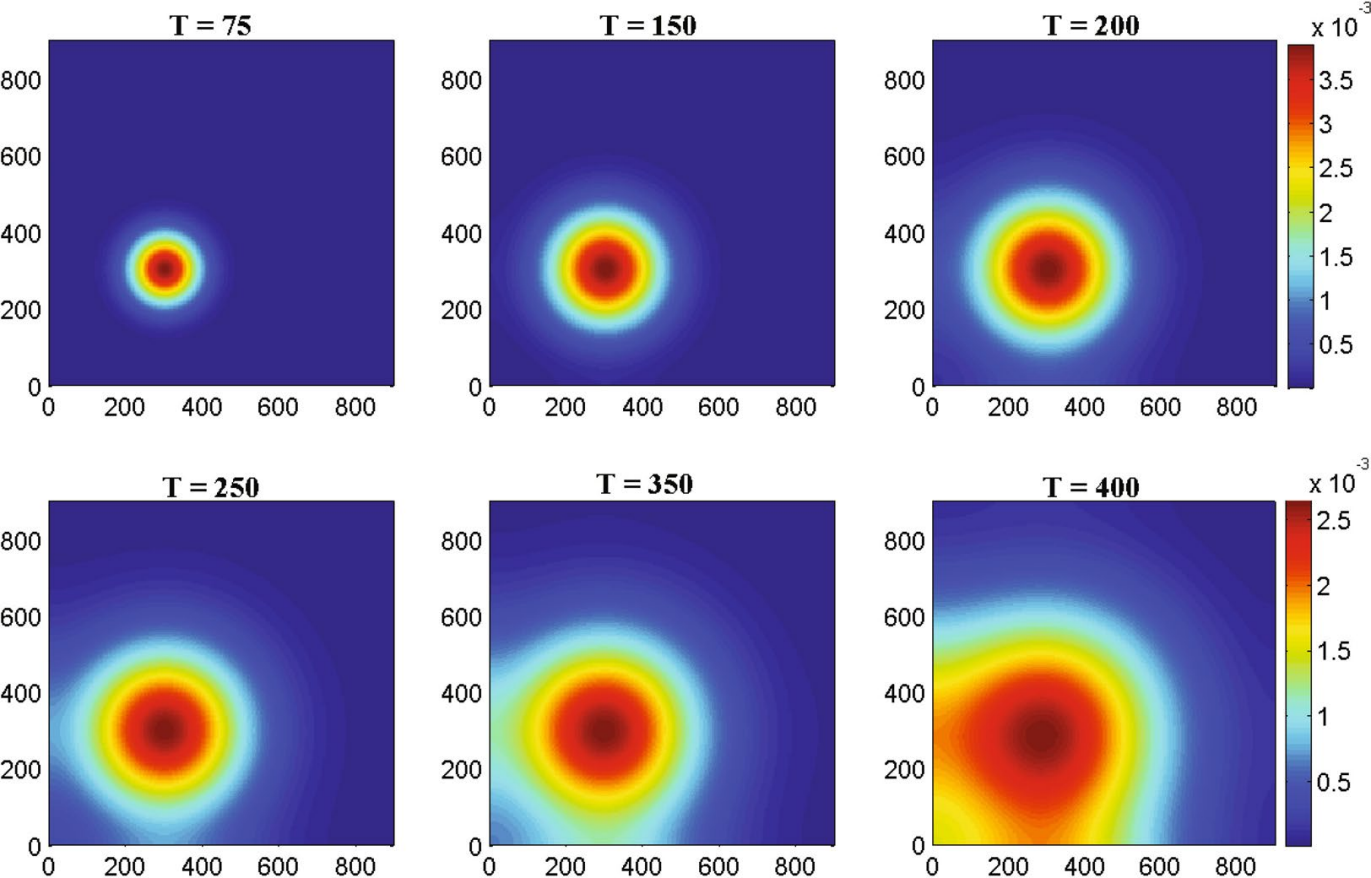
The two-dimensional numerical simulations describe the dynamics of cytotoxic T-lymphocytes cells density $C_{T}(t, x, y)$ at times corresponding to 75, 150, 200, 250, 350 and 400 h respectively. The simulation demonstrates the spatial distribution of cytotoxic T-lymphocytes density through the domain for an increasing value of time. Parameter values: $D_{G} = 55$, $D_{M} = 5$, $D_{T} = 400$, $D_{C} = D_{I} = 25$ and the other parameter values and initial conditions are presented in the Table 1.

Figure 8 represents the results of numerical illustrations for the system (1)–(5) in one-dimensional spatiotemporal dynamics of activated cytotoxic T-lymphocytes whereas the comparable two-dimensional spatiotemporal shaded plot has been shown in the Fig. 9 under different time scenarios. Figure 8 exhibits the one-dimensional spatiotemporal kinetics of cytotoxic T-lymphocytes within the brain domain corresponding to generation times 75, 150, 200, 250, 350 and 400 h respectively. The migration of malignant gliomas has been shown in the Figs. 4 and 5, which shows similar type of patterns in the Figs. 8 and 9 respectively for cytotoxic T-lymphocytes. From the figures it is clear that the spread of migration for malignant gliomas (Figs. 4, 5) is faster than the migration of cytotoxic T-lymphocytes (Figs. 8, 9) as the recruitment of cytotoxic T-lymphocytes depends on the direct presence of malignant gliomas, where the parameter $a_{2}$ models the antigenicity of the brain tissue. Antigenicity can be considered as a measure of how different the malignant gliomas is from ‘self’.

In case of two-dimensional domain, the patterns of malignant gliomas has been demonstrated in the Fig. 5 under different values of time and the patterns of activated cytotoxic T-lymphocytes has been demonstrated in the Fig. 9 with the same values of time. After careful observation, it can be observed that the malignant gliomas spread almost half of the brain domain at time $T =$ 350 h ($\sim$14 days) but for the same time [at $T =$ 350 h, ($\sim$14 days)] the migration of cytotoxic T-lymphocytes is moderate. However, the observed patterns are not uniform in shape (alter temporally) and therefore, their behavior is reported as quasi-stationary. The sixth sub-figure (see the Figs. 5 and 9) highlights the kinetics of the malignant glioma cells and cytotoxic T-lymphocytes, spread almost the major portion of the domain (time *T* = 400 h; $\sim$17 days). The most fascinating characteristic is that the spatiotemporal pattern or the spread of cells is non-monotone and their pattern changes over time.

**Figure 10:**
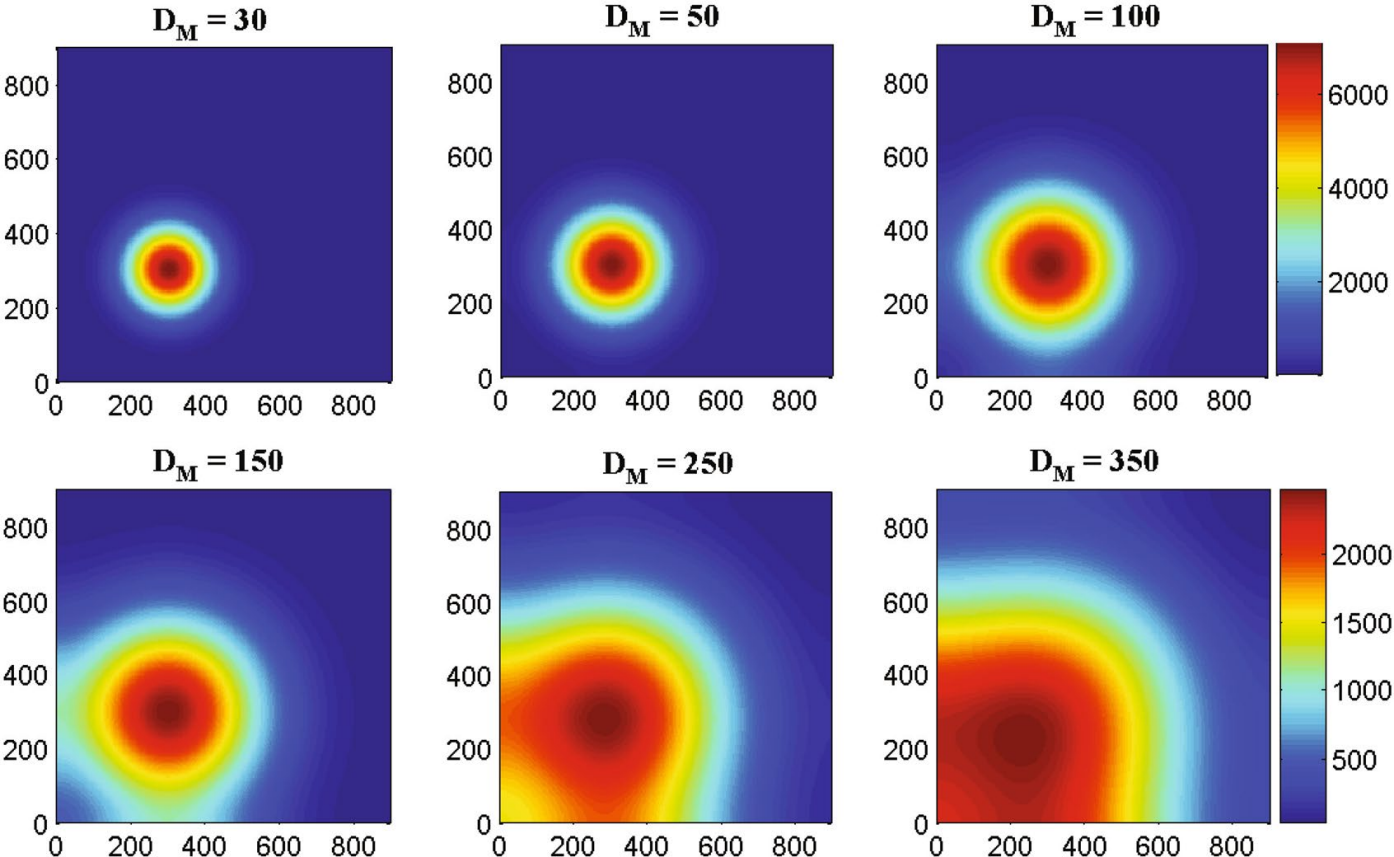
The two-dimensional sequence of profiles designates the evolution of the macrophages density *M*(*t*, *x*, *y*) at time $T = 100~hrs (\sim 4~ days)$ and for different rate of diffusion of macrophages corresponding to 30, 50, 100, 150, 250 and 350 respectively. The simulations shows the spread of macrophages density for an increasing value of the rate of random motility. Parameter values: $D_{G} = 55$, $D_{T} = 400$, $D_{C} = D_{I} = 25$ and the other parameter values and initial conditions are presented in the Table 1.

Now, we shall investigate how different random motility rate $D_{M}$ of macrophages influence the dynamics of macrophages population at the generation time $T =$ 100 h ($\sim$4 days). The two-dimensional Fig. 10 represents the spatiotemporal patterns of macrophages at time $T = 100$ h with different diffusion rates. Figure 10 demonstrates the sensitivity of diffusion coefficient of macrophages due to pseudopodia-mediated migration; the other cells are not so sensitive with the rate of random motility which has not been shown in this figure. From the Fig. 10, it can be observed that at $T =$ 100 h ($\sim$4 days) the macrophages are small in size with $D_{M} = 30$ but for the increased value of random motility rate $D_{M} = 100$ the macrophages are spread quickly throughout the domain. At $D_{M} = 250$, the macrophages are spread almost half of the domain and at $D_{M} = 350$ the macrophages are spread in the left-hand of the domain. In this regard we may assume that the left-hand boundary constitutes a zone of hard bone or tissue that the macrophages are impotent to overcome (due to zero-flux boundary conditions) and hence they begin to move in the opposite direction, driven mainly by chemotaxis^53^ and pseudopodia-mediated migration.

## Discussion

In the present paper we studied a spatiotemporal model which depicts the growth and invasion of glioma cell population and immune system responses based on our previous work^16^. The present work focused on the interaction of glioma cell population, macrophages and activated cytotoxic T-lymphocytes through a system of reaction-diffusion equations. The numerical simulation of the model shows that for an increasing value of time, the glioma cell population, macrophages and cytotoxic T-lymphocytes spread throughout the domain. The reaction-diffusion system has been recognized as an important tool for replying questions relating to proliferations, invasions, migrations and the spatiotemporal pattern formation. Our primary goal was to appraise the viable impacts for glioma invasion in a multi-species community and to investigate the existence of inhomogeneous pattern formation during the interaction with other immune components as well as cytokines. Basically, the existence of spatiotemporal heterogeneity demonstrates that dispersive glioma-immune interactions will exhibit the spatial variation, which has been reported in the existing literatures^15,22,23,55^. Our proposed model underscores the broad range of kinetics which appear from the interaction of spatial random motility and heterogeneous dynamics in a glioma-immune system interactions.

Gliomas are difficult to treat due to its infiltrative behavior, heterogeneity, isolated position beyond the blood-brain-barrier and their migration. Thus, it is very essential to investigate the proliferation/migration pattern of glioma cell population; such investigations arise in Kim et al.^22^, Swanson et al.^5,20^, Harpold et al.^25^. In our study, we investigated the invasion of malignant gliomas and the reasons by which the immune components are spreading with respect to the time and space. The investigated heterogeneous dynamics appear due to coupling of random motility^14,35,58^. There is a corroboration for the occurrence of dense cluster of macrophages, whose orientation is thought to be interconnected to glioma-cytotoxic T-lymphocytes effect of macrophages. This confirms our observation that the interaction among macrophages and malignant glioma cell population is a developing procedure for producing spatial heterogeneity.

The migration of glioma cell population from the primary brain tumor depends both on the glioma cell line and on the glioma micro-environment^22^. In our study, we derive the reaction-diffusion equations to model the spread of glioma-immune system interaction within the brain tissue which is considered as spatially homogeneous medium. Due to the migration of glioma micro-environment, we have tried to comprehend by using reaction-diffusion modeling how glioma cell population and immune components spread over the domain and how this migration evolves in response with various crucial parameters. The first parameter, the macrophages diffusion coefficient $D_{M}$, is highly related to the migration of cells. In the “Qualitative study the model”, we performed the local stability analysis in both the temporal and spatiotemporal system. We employed dispersion relation in the same section and it becomes clear that after the inhomogeneous perturbations, the model system is stable forever. We observed that the spread of macrophages (or macrophage cell distribution) are more sensitive to the rate of random motility of macrophages population. It is to be noted that the irregular spatiotemporal pattern formation of immune system response has been studied by Owen and Sherratt^15^ by considering macrophage interactions with mutant/tumor cells. Our results for macrophages interaction with malignant glioma cell population has much in common with Owen and Sherratt’s^15^ observations. It is worth mentioning that the gliomas, macrophages and cytotoxic T-lymphocytes are more sensitive to the time which has been shown in the numerical simulations. Our spatial aspects in glioma-immune system dynamics provide a fine resemblance between our prognosis at the temporal and spatiotemporal scales.

The numerical illustrations of the proposed model make it easier to understand the biological frameworks associated in the existence of spatiotemporal irregularities observed in the growth of malignant gliomas, macrophages and activated CD8+T cells, which has been reported in various immunomorphological explorations^55^. Meanwhile, in this paper we observed some of the important heterogeneous features interconnected with the mathematical theory of interplays among malignant gliomas, macrophages and cytotoxic T-lymphocytes. We also observed that for an increasing value of time, the glioma cell population, macrophages and cytotoxic T-lymphocytes spread throughout the domain.

The results appear in this article indicating the additional observation of glioma-immune system interactions through a coupled system of reaction-diffusion equations. Basically, an explicit two-dimensional (space) model can be considered, facilitating us to explore the role of asymmetry. Webb et al.^60^ developed a mathematical population model for tumor cell encounter against cytotoxic T-cells to study the effect of asymmetrical behavior. There are significant number of unanswered questions regarding multi-cellular glioma-immune system interactions in a spatiotemporal setting. Closer surveillance has to be paid for parametrization to form the mathematical model and to better understand the dynamics. The model studied in this manuscript, we have considered the medium is homogeneous. Our selection of square domains could also prevent us from predicting more diverse patterns. Diverse migration patterns might appear if the domain for different cells is not square. We hope that these situations would be addressed in our future work. We believe that the investigations presented in this paper will provide a better concept for the clinicians and oncologists to understand the complex dynamics of glioma-immune interactions and to design more efficacious treatment strategies to control and eradicate the glioma cell population. In the present study we have not considered the treatment strategies to control the spread of malignant glioma cell population. Thus, the treatment strategy will be part of our future research.

## Supplementary Information

Supplementary Information.

## Data Availability

All relevant data are within the paper.
