# Supplementary material for: Spatiotemporal dynamics of a glioma immune interaction model
Source: Sci Rep. 2021 Nov 17;11:22385. doi: 10.1038/s41598-021-00985-1 (PMC8599515; doi:10.1038/s41598-021-00985-1)
Supplement: Supplementary file 1 — Supplementary Information. [file 41598_2021_985_MOESM1_ESM.pdf]

Supplementary Material:

Spatiotemporal dynamics of a gliomas immune interaction model

Subhas Khajanchi[1] and Juan J Nieto[2]

[1] *Department of Mathematics, Presidency University, 86/1 College St., Kolkata 700073, India*

Email: subhas.maths@presiuniv.ac.in

[2] *Instituto de Matematicas, Universidade de Santiago de Compostela, Santiago de Compostela, Spain*

I. NUMERICAL SCHEME

Let $\vec{e} = (G, M, C_T, T_\beta, I_\gamma)^T$ be the solution of the model system under consideration. Then the approximation of the solution $\vec{e}$ in one dimension at each of the grid point (x_i, t_n) is $\vec{E}_i^n = ({G_i}^n, {M_i}^n, {C_{Ti}}^n, {T_{\beta_i}}^n, {I_{\gamma_i}}^n)$. Again in two dimensions at each of the grid point (x_i, y_j, t_n), the approximation of the solution $\vec{e}$ is $\vec{E}_{i,j}^n = ({G_{i,j}}^n, {M_{i,j}}^n, {C_{Ti,j}}^n, {T_{\beta_{i,j}}}^n, {I_{\gamma_{i,j}}}^n)$. We denote F_1, F_2, F_3, F_4 and F_5 are the discrete kinetic functions corresponding to f_1, f_2, f_3, f_4 and f_5, respectively. Then the general form of one dimensional linear scheme is as follows:

$$\begin{aligned}
\partial_n {G_i}^n &= D_G {G_i}^n + F_1(\vec{E}_i^n, \vec{E}_i^{n-1}) \\
\partial_n {M_i}^n &= D_M {M_i}^n + F_2(\vec{E}_i^n, \vec{E}_i^{n-1}) \\
\partial_n {C_{Ti}}^n &= D_C {C_{Ti}}^n + F_3(\vec{E}_i^n, \vec{E}_i^{n-1}) \\
\partial_n {T_{\beta_i}}^n &= D_T {T_{\beta_i}}^n + F_4(\vec{E}_i^n, \vec{E}_i^{n-1}) \\
\partial_n {I_{\gamma_i}}^n &= D_I {I_{\gamma_i}}^n + F_5(\vec{E}_i^n, \vec{E}_i^{n-1})
\end{aligned} \tag{1}$$

with initial approximations:

$${G_i}^0 = G_0(x_i), \quad {M_i}^0 = M_0(x_i), \quad {C_{Ti}}^0 = C_{T0}(x_i), \quad {T_{\beta_i}}^0 = T_{\beta 0}(x_i), \quad {I_{\gamma_i}}^0 = I_{\gamma 0}(x_i),$$

where $n = 0, 1, 2, \ldots, N$ and $i = 0, 1, 2, \ldots, J$.

The general form of two dimensional linear scheme are as follows:

$$\begin{aligned}
\partial_n {G_{i,j}}^n &= D_G {G_{i,j}}^n + F_1(\vec{E}_{i,j}^n, \vec{E}_{i,j}^{n-1}) \\
\partial_n {M_{i,j}}^n &= D_M {M_{i,j}}^n + F_2(\vec{E}_{i,j}^n, \vec{E}_{i,j}^{n-1}) \\
\partial_n {C_{Ti,j}}^n &= D_C {C_{Ti,j}}^n + F_3(\vec{E}_{i,j}^n, \vec{E}_{i,j}^{n-1}) \\
\partial_n {T_{\beta_{i,j}}}^n &= D_T {T_{\beta_{i,j}}}^n + F_4(\vec{E}_{i,j}^n, \vec{E}_{i,j}^{n-1}) \\
\partial_n {I_{\gamma_{i,j}}}^n &= D_I {I_{\gamma_{i,j}}}^n + F_5(\vec{E}_{i,j}^n, \vec{E}_{i,j}^{n-1})
\end{aligned} \tag{2}$$

with initial approximations:

$${G_i}^0 = G_0(x_i, y_j), \quad {M_i}^0 = M_0(x_i, y_j), \quad {C_{Ti}}^0 = C_{T0}(x_i, y_j), \quad {T_{\beta_i}}^0 = T_{\beta 0}(x_i, y_j), \quad {I_{\gamma_i}}^0 = I_{\gamma 0}(x_i, y_j),$$

where $n = 0, 1, 2, \ldots, N$ and $i, j = 0, 1, 2, \ldots, J$. We define

$$\tilde{J} = \begin{cases} J, & \text{if dimension is one} \\ (J+1)^2 - 1, & \text{if dimension is two.} \end{cases} \tag{3}$$

Number of auxiliary conditions that approximate the zero-flux boundary conditions of the continuous equations is needed for both the cases (one dimensional and two dimensional cases). The above system of equations (2) are solved using the following scheme:

The kinetics in one dimension corresponds to the system (1) are

$$
\begin{aligned}
F_1(\vec{E}_i^n, \vec{E}_i^{n-1}) = F_{11}(\vec{E}_i^{n-1}) &= r_1 {G_i}^{n-1} - \frac{r_1}{G_{max}} {G_i}^{n-1} |{G_i}^{n-1}| - \frac{(\alpha_1 {M_i}^{n-1} + \alpha_2 {C_{Ti}}^{n-1}){G_i}^{n-1}}{(e_1 + {T_{\beta_i}}^{n-1})({G_i}^{n-1} + k_1)} \\
F_2(\vec{E}_i^n, \vec{E}_i^{n-1}) = F_{21}(\vec{E}_i^{n-1}) &= r_2 {M_i}^{n-1} - \frac{r_2}{M_{max}} {M_i}^{n-1} |{M_i}^{n-1}| \\
&+ a_1 \left(\frac{{I_{\gamma_i}}^{n-1}}{k_4 + {K_{\gamma_i}}^{n-1}} \right) \left(\frac{1}{{T_{\beta_i}}^{n-1} + e_2} \right) - \alpha_3 \frac{{G_i}^{n-1} {M_i}^{n-1}}{k_2 + {G_i}^{n-1}} \\
F_3(\vec{E}_i^n, \vec{E}_i^{n-1}) = F_{31}(\vec{E}_i^{n-1}) &= a_2 {G_i}^{n-1} \frac{1}{k_5 + {T_{\beta_i}}^{n-1}} - \mu_1 {C_{Ti}}^{n-1} - \alpha_4 \frac{{G_i}^{n-1} {C_{Ti}}^{n-1}}{k_3 + {G_i}^{n-1}} \\
F_4(\vec{E}_i^n, \vec{E}_i^{n-1}) = F_{41}(\vec{E}_i^{n-1}) &= s_1 + b_1 {G_i}^{n-1} - \mu_2 {T_{\beta_i}}^{n-1} \\
F_5(\vec{E}_i^n, \vec{E}_i^{n-1}) = F_{51}(\vec{E}_i^{n-1}) &= b_2 {C_{Ti}}^{n-1} - \mu_3 {I_{\gamma_i}}^{n-1},
\end{aligned} \tag{4}
$$

while in case of two dimensions, the kinetics corresponding to the system (2) are as follows:

$$
\begin{aligned}
F_1(\vec{E}_{i,j}^n, \vec{E}_{i,j}^{n-1}) = F_{11}(\vec{E}_{i,j}^{n-1}) &= r_1 {G_{i,j}}^{n-1} - \frac{r_1}{G_{max}} {G_{i,j}}^{n-1} |{G_{i,j}}^{n-1}| \\
&- \frac{(\alpha_1 {M_{i,j}}^{n-1} + \alpha_2 {C_{Ti,j}}^{n-1}){G_{i,j}}^{n-1}}{(e_1 + {T_{\beta_{i,j}}}^{n-1})({G_{i,j}}^{n-1} + k_1)} \\
F_2(\vec{E}_{i,j}^n, \vec{E}_{i,j}^{n-1}) = F_{21}(\vec{E}_{i,j}^{n-1}) &= r_2 {M_{i,j}}^{n-1} - \frac{r_2}{M_{max}} {M_{i,j}}^{n-1} |{M_{i,j}}^{n-1}| \\
&+ a_1 \left(\frac{{I_{\gamma_{i,j}}}^{n-1}}{k_4 + {K_{\gamma_{i,j}}}^{n-1}} \right) \left(\frac{1}{{T_{\beta_{i,j}}}^{n-1} + e_2} \right) - \alpha_3 \frac{{G_{i,j}}^{n-1} {M_{i,j}}^{n-1}}{k_2 + {G_{i,j}}^{n-1}} \\
F_3(\vec{E}_{i,j}^n, \vec{E}_{i,j}^{n-1}) = F_{31}(\vec{E}_{i,j}^{n-1}) &= a_2 {G_{i,j}}^{n-1} \frac{1}{k_5 + {T_{\beta_{i,j}}}^{n-1}} - \mu_1 {C_{Ti,j}}^{n-1} - \alpha_4 \frac{{G_{i,j}}^{n-1} {C_{Ti,j}}^{n-1}}{k_3 + {G_{i,j}}^{n-1}} \\
F_4(\vec{E}_{i,j}^n, \vec{E}_{i,j}^{n-1}) = F_{41}(\vec{E}_{i,j}^{n-1}) &= s_1 + b_1 {G_{i,j}}^{n-1} - \mu_2 {T_{\beta_{i,j}}}^{n-1} \\
F_5(\vec{E}_{i,j}^n, \vec{E}_{i,j}^{n-1}) = F_{51}(\vec{E}_{i,j}^{n-1}) &= b_2 {C_{Ti,j}}^{n-1} - \mu_3 {I_{\gamma_{i,j}}}^{n-1}
\end{aligned} \tag{5}
$$

Above scheme can be expressed as $2(\tilde{J}+1)$ linear equations as follows:

$$
\begin{bmatrix} \Phi_1 & 0 & 0 & 0 & 0 \\ 0 & \Phi_2 & 0 & 0 & 0 \\ 0 & 0 & \Phi_3 & 0 & 0 \\ 0 & 0 & 0 & \Phi_4 & 0 \\ 0 & 0 & 0 & 0 & \Phi_5 \end{bmatrix}
\begin{bmatrix} \vec{G}^n \\ \vec{M}^n \\ \vec{C_T}^n \\ \vec{T_\beta}^n \\ \vec{I_\gamma}^n \end{bmatrix}
=
\begin{bmatrix} \vec{G}^{n-1} + \Delta t \vec{F}_{12} \\ \vec{M}^{n-1} + \Delta t \vec{F}_{22} \\ \vec{C_T}^{n-1} + \Delta t \vec{F}_{32} \\ \vec{T_\beta}^{n-1} + \Delta t \vec{F}_{42} \\ \vec{I_\gamma}^{n-1} + \Delta t \vec{F}_{52} \end{bmatrix}, \quad n = 1, 2, \ldots, N. \tag{6}
$$

where $\left\{\vec{F}_{12}\right\}_k = F_{11}(\vec{E}_k^{n-1})$, $\left\{\vec{F}_{22}\right\}_k = F_{21}(\vec{E}_k^{n-1})$, $\left\{\vec{F}_{32}\right\}_k = F_{31}(\vec{E}_k^{n-1})$, $\left\{\vec{F}_{42}\right\}_k = F_{41}(\vec{E}_k^{n-1})$, $\left\{\vec{F}_{52}\right\}_k = F_{51}(\vec{E}_k^{n-1})$, for $k = 0, 1, 2, \ldots, \tilde{J}$. The constant coefficient matrices $\Phi_1, \Phi_2, \Phi_3, \Phi_4$ and Φ_5 are defined in the below.

In case of one dimension, Γ matrix is defined as follows:

$$\Gamma = \frac{1}{h^2}\begin{bmatrix} 2 & -2 & & & & & \\ -1 & 2 & -1 & & & & \\ & -1 & 2 & -1 & & & \\ & & \ddots & \ddots & \ddots & & \\ & & & -1 & 2 & -1 & \\ & & & & -1 & 2 & -1 \\ & & & & & -2 & 2 \end{bmatrix}_{(J+1)\times(J+1)}$$

In case of two dimensions, Γ matrix is defined as follows:

$$\Gamma = \frac{1}{h^2}\begin{bmatrix} A & B & & & & & \\ P & Q & P & & & & \\ & P & Q & P & & & \\ & & \ddots & \ddots & \ddots & & \\ & & & P & Q & P & \\ & & & & P & Q & P \\ & & & & & R & S \end{bmatrix}_{(J+1)^2\times(J+1)^2}, A = \begin{bmatrix} 3 & -3/2 & & & & & \\ -1 & 4 & -1 & & & & \\ & -1 & 4 & -1 & & & \\ & & \ddots & \ddots & \ddots & & \\ & & & -1 & 4 & -1 & \\ & & & & -1 & 4 & -1 \\ & & & & & -3 & 6 \end{bmatrix}_{(J+1)\times(J+1)},$$

$B = diag\{-3/2, -2, -2, \ldots, -2, -2, -3\}$,

$P = -I$,

$R = diag\{-3, -2, -2, \ldots, -2, -2, -3/2\}$,

$$Q = \begin{bmatrix} 4 & -2 & & & & & \\ -1 & 4 & -1 & & & & \\ & -1 & 4 & -1 & & & \\ & & \ddots & \ddots & \ddots & & \\ & & & -1 & 4 & -1 & \\ & & & & -1 & 4 & -1 \\ & & & & & -2 & 4 \end{bmatrix}_{(J+1)\times(J+1)}, S = \begin{bmatrix} 6 & -3 & & & & & \\ -1 & 4 & -1 & & & & \\ & -1 & 4 & -1 & & & \\ & & \ddots & \ddots & \ddots & & \\ & & & -1 & 4 & -1 & \\ & & & & -1 & 4 & -1 \\ & & & & & -3/2 & 3 \end{bmatrix}_{(J+1)\times(J+1)}.$$

The coefficient matrix of the linear system (6) are defined as follows:

$$\begin{aligned}\Phi_1 &= I + D_G \Delta t \Gamma,\\ \Phi_2 &= I + D_M \Delta t \Gamma,\\ \Phi_3 &= I + D_C \Delta t \Gamma,\\ \Phi_4 &= I + D_T \Delta t \Gamma,\\ \Phi_5 &= I + D_I \Delta t \Gamma.\end{aligned}$$

II. SIMULATION FRAMEWORK: WORKING PROCEDURE

Step 1. Initialize the model parameters.

Step 2. Initialize the initial functions.

Step 3. Calculate and assign system constants.

Step 4. Set up grid points.

Step 5. Construction of the matrix Γ.

Step 6. Construction of the coefficient matrices $\Phi_1, \Phi_2, \Phi_3, \Phi_4$ and Φ_5.

Step 7. Solve the model system for G, M, C_T, T_β and I_γ in time.

Step 8. Plot the solutions of the proposed model.
